# Dietary index for gut microbiota and risk of incident gastroesophageal reflux disease: a prospective cohort analysis integrating plasma proteomics in the UK Biobank

**DOI:** 10.3389/fnut.2026.1880631

**Published:** 2026-07-07

**Authors:** Yingxuan Huang, Xiaobo Liu, Chanchan Lin, Yingyi Li, Apei Zhou, Yisen Huang, Yubin Wang, Xiaoqiang Liu

**Affiliations:** 1 Department of Gastroenterology, First Hospital of Quanzhou Affiliated to Fujian Medical University, Quanzhou, Fujian, China; 2 McConnell Brain Imaging Centre, Montreal Neurological Institute, McGill University, Montreal, QC, Canada

**Keywords:** dietary index for gut microbiota, gastroesophageal reflux disease, mediation analysis, plasma proteomics, UK Biobank

## Abstract

**Objectives:**

Gastroesophageal reflux disease (GERD) is a common chronic digestive disorder, and diet is an important modifiable risk factor. The Dietary Index for Gut Microbiota (DI-GM) reflects dietary patterns considered favorable to the gut microbiota; however, the gut microbiome itself was not measured, and evidence on whether DI-GM is associated with GERD risk is limited. We aimed to evaluate the association between DI-GM and incident GERD and to explore potential intermediate pathways using mediation analysis and plasma proteomics.

**Methods:**

We included 133,915 UK Biobank participants free of GERD at baseline. DI-GM scores (range 0–14) were calculated from the Oxford WebQ 24-h dietary recall and grouped into four categories (0–3, 4, 5, ≥6). Incident GERD was identified from the UK Biobank first-occurrence records (ICD-10 K21). Cox proportional hazards regression was complemented by restricted cubic spline (RCS), subgroup, and Cox weighted quantile sum (WQS) analyses, counterfactual mediation analysis, integrated plasma proteomics (Olink Explore 3072), and a range of sensitivity analyses.

**Results:**

During a median follow-up of 13.6 years, 12,271 incident GERD cases occurred. In the fully adjusted model, each 1-point increase in DI-GM was associated with an approximately 4% lower hazard of GERD [hazard ratio (HR) 0.956, 95% CI 0.947–0.965; *P* < 0.001], and the highest DI-GM group (≥6) had a lower hazard than the lowest (0–3) (HR 0.812, 95% CI 0.774–0.851; *P* for trend < 0.001). RCS analysis showed a modest inverse dose–response relationship that was most apparent at higher DI-GM scores, and WQS analysis indicated that the association was driven by a few key components rather than shared equally across the index. BMI (proportion mediated 21.43%) and phenotypic age acceleration (7.53%) both significantly mediated the association (both *P* < 0.001). Integrated proteomic analysis identified 13 shared proteins; the nine positively associated with DI-GM and inversely associated with GERD showed exploratory enrichment in neurodevelopment- and cell-adhesion-related processes.

**Conclusions:**

Higher DI-GM was associated with a lower incidence of medically attended GERD, with mediation analysis suggesting that BMI and biological aging may partly account for this association. Exploratory proteomic analyses identified shared protein correlates, including adiposity-related and, tentatively, neurodevelopment- and cell-adhesion-related proteins; these are hypothesis-generating and require confirmation. Because the gut microbiome was not measured, the diet–microbiota link remains inferential, and these findings should be interpreted as associations rather than established mechanisms.

## 1 Introduction

Gastroesophageal reflux disease (GERD) is one of the most common chronic digestive disorders worldwide, with an estimated global prevalence of approximately 13% in adults and a continuing upward trend (1). GERD substantially impairs quality of life and is closely linked to esophagitis, Barrett's esophagus, and esophageal adenocarcinoma, contributing to a growing medical burden (2). Identifying modifiable risk factors for GERD is therefore of considerable public health importance.

Diet is considered one of the most important modifiable risk factors for GERD (3). Most previous studies, however, have focused on individual foods or nutrients and have struggled to capture overall dietary patterns at the population level (4). Emerging evidence has implicated the gut microbiota in GERD pathogenesis, potentially through effects on gastrointestinal motility, mucosal barrier integrity, and bidirectional signaling along the gut–brain–esophageal axis, with diet being a major environmental factor shaping gut microbial composition; this has motivated the hypothesized “diet–gut microbiota–GERD” pathway (5, 6). Several of these mechanisms are proximate to reflux pathophysiology rather than markers of general diet quality, and could influence reflux independently of socioeconomic position; a microbiota-favorable diet may additionally lower adiposity and systemic low-grade inflammation, thereby reducing intra-abdominal pressure and stress on the lower esophageal sphincter (7–9).

To capture the influence of habitual diet on the gut microbiota at the population level, Kase et al. (10) proposed the Dietary Index for Gut Microbiota (DI-GM) in 2024, which integrates 14 dietary components classified as either beneficial or unfavorable to the gut microbiota based on existing evidence. It should be emphasized, however, that DI-GM is a dietary proxy for a microbiota-favorable pattern: the gut microbiome itself is not measured, and any diet–microbiota link inferred from the index therefore remains indirect. Since its introduction, higher DI-GM scores have been associated with lower risks of several metabolic and psychiatric conditions (11–13); however, the association between DI-GM and incident GERD has not been systematically evaluated in a large prospective cohort, and its potential intermediate pathways remain unclear. High-throughput plasma proteomics, such as the Olink platform, offers an opportunity to explore plasma protein correlates of DI-GM that are also associated with GERD. We therefore undertook an exploratory, hypothesis-generating proteomic analysis to identify candidate proteins and pathways, recognizing that such analyses generate hypotheses for future testing rather than confirm pre-specified mechanisms.

Against this background, the present study uses the UK Biobank prospective cohort to evaluate the association between DI-GM and its beneficial and unfavorable sub-scores with incident GERD, to identify the key dietary components within the DI-GM that contribute most to this association, to examine whether body mass index (BMI) and phenotypic age acceleration mediate this association, and to integrate plasma proteomic data to identify shared proteins and candidate biological pathways linking DI-GM to GERD, with the aim of providing epidemiological and exploratory molecular evidence on the association between a microbiota-relevant dietary pattern and incident GERD, rather than to establish causation.

## 2 Materials and methods

### 2.1 Study design and participants

This was a prospective cohort study based on the UK Biobank, a large population-based cohort that recruited approximately 500,000 participants aged 40–69 years from 22 assessment centers across England, Scotland and Wales between 2006 and 2010 (14). At baseline, all participants completed touchscreen questionnaires, face-to-face interviews, physical measurements, and biological sample collection, and provided written informed consent. The UK Biobank study was approved by the North West Multi-Centre Research Ethics Committee (reference 21/NW/0157). The present analysis was conducted under UK Biobank application number 1099827.

**Figure 1:**
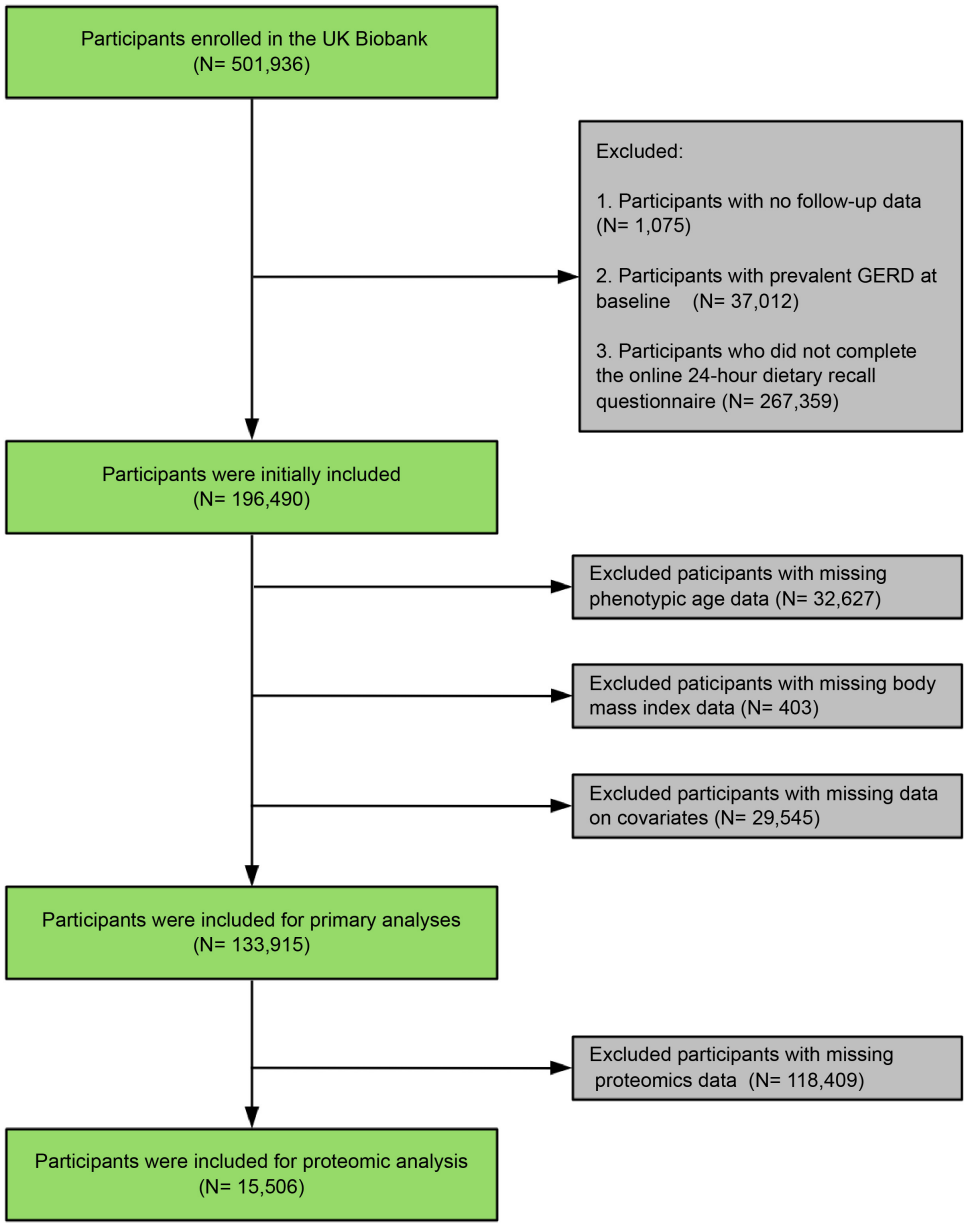
Participant inclusion flowchart.

Of 501,936 participants initially enrolled, we sequentially excluded participants without follow-up data (*n* = 1,075), those with prevalent GERD at baseline (*n* = 37,012), and those who had not completed the Oxford WebQ 24-h dietary recall (*n* = 267,359), yielding an initial sample of 196,490 participants. We further excluded those with missing data on phenotypic age (*n* = 32,627), BMI (*n* = 403), and other key covariates (*n* = 29,545), resulting in a final analytical sample of 133,915 participants. For proteomic analyses, an additional 118,409 participants without baseline plasma proteomic data were excluded, yielding 15,506 participants (Figure 1).

### 2.2 Dietary index for gut microbiota (DI-GM)

The DI-GM was originally proposed by Kase et al. in 2024 to quantify the favorability of habitual dietary intake for gut microbiota diversity and composition. The index comprises 14 dietary components grouped into two directional categories. 10 components are considered beneficial to the gut microbiota, including avocado, broccoli, chickpeas, coffee, cranberries, fermented dairy, dietary fiber, green tea, soybeans, and whole grains. Four components are considered unfavorable to the gut microbiota: red meat, processed meat, refined grains, and a high-fat dietary pattern (defined as ≥40% of total energy intake from fat). Detailed food component definitions and calculation procedures are provided in Supplementary Table 1.

Dietary intake was assessed using the Oxford WebQ 24-h dietary recall questionnaire. For participants who completed multiple recalls, the mean of all available recalls was used to represent habitual intake. Among the 133,915 participants in the main analytic cohort, the number of completed Oxford WebQ recalls was distributed as follows: one recall, 51,575 (38.5%); two recalls, 30,969 (23.1%); three recalls, 27,678 (20.7%); four recalls, 19,947 (14.9%); and five recalls, 3,746 (2.8%). Each component was scored dichotomously using sex-specific medians as the cut-off: beneficial components scored one point if intake was at or above the sex-specific median (zero otherwise), and unfavorable components scored one point if intake was below the median, with the high-fat pattern scored using the predefined 40% threshold. The total DI-GM score (range 0–14) was the sum of all 14 components, with higher scores indicating a more microbiota-favorable diet. Two sub-scores were derived: a beneficial component score (range 0–10) and an unfavorable component score (range 0–4). DI-GM was further categorized into four groups (0–3, 4, 5, and ≥6), following the categorization applied in a previous DI-GM study (15); these category boundaries corresponded to the empirical quartile cut-points (the 25th, 50th, and 75th percentiles) of the score distribution in the analytic cohort.

### 2.3 Ascertainment of incident GERD

The primary outcome was incident GERD. GERD events were identified from the UK Biobank First Occurrences data using the ICD-10 code K21 for gastroesophageal reflux disease (16). Specifically, GERD events were defined according to UK Biobank Data-Field 131585 [“Source of report of K21 (gastroesophageal reflux disease)”] and its corresponding date-of-first-occurrence field. This First Occurrences variable integrates outcome information from multiple sources, including hospital inpatient records, primary care records, death registry data, and self-report. Participants with any K21-related record or self-reported GERD before baseline were classified as prevalent cases and excluded. Follow-up began at the baseline assessment date and continued until the earliest of first GERD diagnosis, death, loss to follow-up, or the administrative censoring date, which differed by region according to record availability: 31 March 2023 for England, 31 August 2022 for Scotland, and 31 May 2022 for Wales.

### 2.4 Covariates

Covariates were selected *a priori* on the basis of established literature and clinical relevance (17, 18). Demographic covariates included age (continuous, years), sex (male/female), and ethnicity (white/other). Socioeconomic covariates included the Townsend deprivation index (continuous, with higher values indicating greater deprivation) and educational attainment (college or university degree vs. other). Lifestyle covariates included smoking status (never/previous/current), alcohol consumption status (never/previous /current), and adherence to the World Health Organization recommendation for moderate-to-vigorous physical activity (MVPA; ≥150 min of moderate or ≥75 min of vigorous activity per week; yes/no) (19). Prevalent comorbidities included cardiovascular disease (CVD), diabetes, hypertension, anxiety, depression, and sleep disorder, ascertained from linked ICD-10 records. Detailed definitions and ascertainment of covariates are provided in Supplementary Table 2.

BMI (kg/m^2^) and phenotypic age acceleration were treated as candidate mediators in the mediation analyses rather than as confounders in the primary models. Phenotypic age was calculated from chronological age and nine clinical biomarkers (albumin, creatinine, glucose, C-reactive protein, lymphocyte percentage, mean corpuscular volume, red cell distribution width, alkaline phosphatase, and white blood cell count) using the algorithm proposed by Levine et al. (20). To capture biological aging independent of chronological age, phenotypic age acceleration was then derived as the residual from a linear regression of phenotypic age on chronological age within the analytic cohort; positive values indicate that an individual is biologically older than expected for their chronological age, and negative values indicate the converse (21). This residual was used as the mediator in all mediation analyses involving biological aging.

### 2.5 Plasma proteomic measurement

Plasma proteomic data were obtained from the UK Biobank Pharma Proteomics Project (UKB-PPP), which used the Olink Explore 3072 platform to measure 2,923 unique proteins (22). Protein levels were expressed as normalized protein expression (NPX) values on a log_2_ scale. Proteins with more than 20% missing values were excluded, and remaining missing values were imputed using the mean value of each protein, yielding 2,910 proteins for downstream analysis (Supplementary Table 3).

### 2.6 Statistical analysis

Baseline characteristics were summarized by DI-GM group using mean ± standard deviation or median (interquartile range) for continuous variables and counts (percentages) for categorical variables. Between-group comparisons were performed using one-way analysis of variance, the Kruskal–Wallis test, or the chi-squared test, as appropriate. The primary analysis examined the association between DI-GM and incident GERD using Cox proportional hazards regression, with DI-GM treated as a continuous variable, a four-level categorical variable, and decomposed into its beneficial and unfavorable sub-scores. Two sequentially adjusted models were constructed: Model 1 adjusted for age and sex; Model 2 additionally adjusted for ethnicity, Townsend deprivation index, education, smoking, alcohol, MVPA, and prevalent CVD, diabetes, hypertension, anxiety, depression, and sleep disorder. The proportional hazards assumption was assessed using scaled Schoenfeld residuals, both for individual covariates and as a global test, with *P* > 0.05 considered consistent with the assumption. Hazard ratios (HRs) and 95% confidence intervals (CIs) were calculated with the lowest DI-GM group (0–3) as the reference, and a trend test was conducted by assigning the median of each group as a continuous variable in the regression. Dose–response relationships between DI-GM-related scores and GERD risk were further evaluated using restricted cubic splines (RCS) with four knots at the 5th, 35th, 65th, and 95th percentiles of the score distribution, with reference points set at the median of each score (5 for total DI-GM, 3 for the beneficial sub-score, and 2 for the unfavorable sub-score). Non-linearity was assessed by likelihood ratio testing. Because the unfavorable sub-score takes only a small number of discrete integer values, the linear estimate from the Cox model was regarded as the primary dose–response inference for this sub-score, with the corresponding RCS interpreted as exploratory. Cumulative incidence curves were generated using the Kaplan–Meier method and compared using the log-rank test. Pre-specified subgroup analyses were performed across strata defined by age (< 65 vs. ≥65 years), sex, smoking, alcohol, MVPA, and prevalent CVD, diabetes, hypertension, anxiety, depression, and sleep disorder. Within each subgroup, models adjusted for all covariates except the stratifying variable; interactions were assessed by likelihood ratio testing. To quantify the relative contribution of individual DI-GM components, we fitted a Cox weighted quantile sum (WQS) regression model. Each component entered the model as its DI-GM component score, already oriented so that a higher score denotes a more microbiota-favorable intake; a weighted index was then constructed with the constraint that the component weights summed to one. The WQS index was then entered into a Cox model adjusted for all covariates, and the stability of estimated weights was evaluated using 1,000 bootstrap resamples.

Counterfactual mediation analyses were conducted to evaluate whether BMI and phenotypic age acceleration mediated the DI-GM–GERD association. The mediation models used an accelerated failure time (AFT) outcome model; accordingly, the total effect, average causal mediation effect (ACME), and average direct effect (ADE) are expressed on the time-to-event scale, as differences in model-predicted time (in years) to incident GERD, with positive values indicating a protective association. Total effects, ACME, ADE, and the proportion mediated were estimated, with 95% CIs derived from 1,000 bootstrap resamples. To avoid over-adjustment of the mediated pathway, the mediation models were adjusted for age, sex, ethnicity, Townsend deprivation index, education, smoking, alcohol, and MVPA, but not for prevalent CVD, diabetes, hypertension, anxiety, depression, or sleep disorder, as these conditions plausibly lie on the causal pathway downstream of the proposed mediators. For the proteomic analyses, multivariable linear regression was used to assess associations between DI-GM and each plasma protein, adjusting for all covariates. Multiple testing was controlled using both Bonferroni correction and the Benjamini–Hochberg false discovery rate (FDR); Bonferroni-significant proteins were used to report the most stringent threshold, while FDR-significant proteins were carried forward for shared-protein identification. Cox proportional hazards models were used to assess associations between each plasma protein (per one standard deviation increase) and incident GERD, adjusting for all covariates with FDR correction. Proteins reaching FDR significance in both the DI-GM–protein and protein–GERD analyses were defined as shared proteins. The subset of shared proteins positively associated with DI-GM and inversely associated with GERD was subjected to Gene Ontology biological process (GO:BP) enrichment analysis using clusterProfiler, with Benjamini–Hochberg-adjusted *P* < 0.05 considered significant.

We performed a series of sensitivity analyses to assess the robustness of the primary findings. First, to reduce exposure misclassification arising from reliance on a single 24-h dietary recall, we repeated the primary Cox analyses restricted to participants who had completed two or more dietary recalls (*n* = 82,340), in whom usual intake is characterized more precisely. Second, to limit the influence of reverse causation, we conducted a lag analysis excluding participants who developed incident GERD within the first 2 years of follow-up. Third, to address potential immortal time bias arising from the interval between the baseline assessment and completion of the Oxford WebQ dietary questionnaire, we re-anchored the start of follow-up to the date of each participant's first completed WebQ and excluded those with GERD recorded before that date (*n* = 131,641). Fourth, to evaluate confounding by overall dietary intake, we additionally adjusted Model 2 for total energy intake. Fifth, because the global Schoenfeld test indicated departures from the proportional hazard's assumption attributable to prevalent depression and sleep disorder, we refitted Model 2 stratified by these two covariates, allowing their baseline hazards to vary while retaining all remaining covariates as adjustments. Sixth, to account for the competing risk of death, we estimated subdistribution hazard ratios using the Fine–Gray model, with death before incident GERD treated as a competing event. Seventh, to gauge robustness to unmeasured confounding, particularly unmeasured use of over-the-counter acid-suppressant medication, we calculated E-values for both the point estimate and the confidence-interval limit nearer the null for the highest vs. lowest DI-GM category.

For the proteomic analyses, we undertook three additional robustness checks. First, to assess the impact of missing-value imputation, we repeated the DI-GM–protein and protein–GERD regressions using complete-case data without imputation. Second, to evaluate the representativeness of the proteomic subsample, we compared its baseline characteristics with those of the full analytic cohort using standardized mean differences. Third, to examine the robustness of the enrichment findings, we repeated the GO:BP enrichment analysis under three alternative protein input sets: all shared proteins without directional pre-selection; the directionally concordant proteins (positively associated with DI-GM and inversely associated with GERD); and the directionally discordant proteins. We then assessed whether the observed number of shared proteins exceeded chance expectation using a hypergeometric test. All analyses were performed using R version 4.3.0 (R Foundation for Statistical Computing, Vienna, Austria) and Python, employing the survival, rms, survminer, gWQS, mediation, cmprsk, and EValue packages; proteomic enrichment analysis was conducted with clusterProfiler. Two-sided *P* < 0.05 was considered statistically significant for primary analyses, with Bonferroni- or FDR-corrected thresholds applied where indicated.

## 3 Results

### 3.1 Baseline characteristics of the study population

After applying the inclusion and exclusion criteria, 133,915 UK Biobank participants were included in the present analysis (Figure 1). The mean baseline age was 55.7 ± 8.0 years, and 70,770 (52.8%) were women. Participants were divided into four DI-GM groups (0–3, 4, 5, and ≥6), comprising 28,495, 25,024, 26,899, and 53,497 individuals, respectively. The mean DI-GM score was 5.1 ± 1.9, with a median beneficial sub-score of 3.0 (IQR 2.0–4.0) and a mean unfavorable sub-score of 2.2 ± 0.9. Baseline characteristics across DI-GM groups are summarized in Table 1. Participants with higher DI-GM scores tended to be older, more frequently male and of white ethnicity, and more likely to hold a college or university degree and to meet recommended MVPA levels (all *P* < 0.001). Conversely, current smoking, higher BMI, and prevalent diabetes, hypertension, and depression were less common in the highest DI-GM group (all *P* < 0.001). No appreciable between-group differences were observed for prevalent CVD, anxiety, or sleep disorder (*P* ≥ 0.058). Phenotypic age increased modestly across higher DI-GM groups, paralleling the older chronological age in these strata; however, phenotypic age acceleration was progressively lower at higher DI-GM, consistent with slower biological aging.

**Table 1:** Baseline characteristics of participants across DI-GM group.

Variables	Total	DI-GM group				*P* value
		0–3	4	5	≥6	
**Number of participants**	133,915	28,495	25,024	26,899	53,497	
**Age, Mean** **±SD**	55.7 ± 8.0	54.2 ± 8.1	55.3 ± 8.0	55.8 ± 8.0	56.7 ± 7.8	< 0.001
**Sex**, ***n*** **(%)**						< 0.001
Female	70,770 (52.8)	16,320 (57.3)	13,408 (53.6)	14,062 (52.3)	26,980 (50.4)	
Male	63,145 (47.2)	12,175 (42.7)	11,616 (46.4)	12,837 (47.7)	26,517 (49.6)	
**Ethnicity**, ***n*** **(%)**						0.003
White	128,576 (96.0)	27,255 (95.6)	2,4019 (96.0)	25,855 (96.1)	51,447 (96.2)	
Others	5,339 (4.0)	1,240 (4.4)	1,005 (4.0)	1,044 (3.9)	2,050 (3.8)	
**Townsend deprivation index, Median (IQR)**	−2.4 (−3.8, 0.0)	−2.2 (−3.7, 0.3)	−2.4 (−3.8, 0.0)	−2.4 (−3.8,−0.1)	−2.4 (−3.8, −0.1)	< 0.001
**Education**, ***n*** **(%)**						< 0.001
College or University degree	61,237 (45.7)	10,657 (37.4)	10,539 (42.1)	12,129 (45.1)	27,912 (52.2)	
Others	72,678 (54.3)	17,838 (62.6)	14,485 (57.9)	14,770 (54.9)	25,585 (47.8)	
**Smoking status, n (%)**						< 0.001
Never	76,116 (56.8)	15,772 (55.4)	14,165 (56.6)	15,345 (57.0)	30,834 (57.6)	
Previous	47,374 (35.4)	9,611 (33.7)	8,672 (34.7)	9,511 (35.4)	19,580 (36.6)	
Current	10,425 (7.8)	3,112 (10.9)	2,187 (8.7)	2,043 (7.6)	3,083 (5.8)	
**Alcohol status, n (%)**						< 0.001
Never	3,890 (2.9)	953 (3.3)	745 (3.0)	752 (2.8)	1,440 (2.7)	
Previous	3872 (2.9)	788 (2.8)	706 (2.8)	731 (2.7)	1,647 (3.1)	
Current	126,153 (94.2)	26,754 (93.9)	23,573 (94.2)	25,416 (94.5)	50,410 (94.2)	
**Meets MVPA recommendation, n (%)**						< 0.001
No	60,848 (45.4)	14,618 (51.3)	12,012 (48.0)	12,279 (45.6)	21,939 (41.0)	
Yes	73,067 (54.6)	13,877 (48.7)	13,012 (52.0)	14,620 (54.4)	31,558 (59.0)	
**CVD, n (%)**						0.742
No	127,724 (95.4)	27,144 (95.3)	23,866 (95.4)	25,666 (95.4)	51,048 (95.4)	
Yes	6,191 (4.6)	1,351 (4.7)	1,158 (4.6)	1,233 (4.6)	2,449 (4.6)	
**Diabetes, n (%)**						< 0.001
No	128,690 (96.1)	27,265 (95.7)	23,998 (95.9)	25,818 (96.0)	51,609 (96.5)	
Yes	5,225 (3.9)	1,230 (4.3)	1,026 (4.1)	1,081 (4.0)	1,888 (3.5)	
**Hypertension, n (%)**						< 0.001
No	102,730 (76.7)	21,570 (75.7)	19,003 (75.9)	20,576 (76.5)	41,581 (77.7)	
Yes	31,185 (23.3)	6,925 (24.3)	6,021 (24.1)	6,323 (23.5)	1,1916 (22.3)	
**Anxiety, n (%)**						0.058
No	129,207 (96.5)	27,421 (96.2)	24,142 (96.5)	25,972 (96.6)	51,672 (96.6)	
Yes	4,708 (3.5)	1,074 (3.8)	882 (3.5)	927 (3.4)	1,825 (3.4)	
**Depression, n (%)**						< 0.001
No	123,787 (92.4)	26,112 (91.6)	23,101 (92.3)	24,916 (92.6)	49,658 (92.8)	
Yes	10,128 (7.6)	2,383 (8.4)	1,923 (7.7)	1,983 (7.4)	3,839 (7.2)	
**Sleep disorder**, ***n*** **(%)**						0.394
No	131,652 (98.3)	27,987 (98.2)	24,591 (98.3)	26,448 (98.3)	52,626 (98.4)	
Yes	2,263 (1.7)	508 (1.8)	433 (1.7)	451 (1.7)	871 (1.6)	
		**0–3**	**4**	**5**	≥**6**	
**DI-GM score, Mean** **±SD**	5.1 ± 1.9	2.5 ± 0.7	4.0 ± 0.0	5.0 ± 0.0	7.0 ± 1.1	< 0.001
**Beneficial to gut microbiota, Median (IQR)**	3.0 (2.0, 4.0)	1.0 (0.0, 2.0)	2.0 (1.0, 3.0)	3.0 (2.0, 3.0)	4.0 (4.0, 5.0)	< 0.001
**Unfavorable to gut microbiota, Mean** **±SD**	2.2 ± 0.9	1.5 ± 0.7	2.0 ± 0.8	2.2 ± 0.8	2.6 ± 0.8	< 0.001
**BMI, Mean** **±SD**	26.8 ± 4.5	27.5 ± 4.9	27.2 ± 4.6	26.8 ± 4.5	26.2 ± 4.2	< 0.001
**Phenotypic age, Mean** **±SD**	56.9 ± 9.3	56.1 ± 9.6	56.7 ± 9.4	57.0 ± 9.4	57.3 ± 9.1	< 0.001
**Phenotypic age acceleration, Median (IQR)**	0.6 (−1.9, 3.4)	1.2 (−1.3, 4.2)	0.8 (−1.7, 3.6)	0.5 (−1.9, 3.4)	0.2 (−2.2, 2.8)	< 0.001

DI-GM, dietary index for gut microbiota; BMI, body mass index; MVPA, moderate-to-vigorous physical activity; CVD, cardiovascular disease.

### 3.2 Association between DI-GM and incident GERD

During a median follow-up of 13.6 years, 12,271 incident GERD cases were identified from the UK Biobank first-occurrence records. Scaled Schoenfeld residuals confirmed that DI-GM satisfied the proportional hazards assumption (χ^2^ = 1.085, *P* = 0.298; Supplementary Figure 1), supporting the validity of the Cox model. In the age- and sex-adjusted Model 1, each 1-point increment in DI-GM was associated with an approximately 6% lower hazard of incident GERD (HR 0.942, 95% CI 0.933–0.951; *P* < 0.001). The association attenuated modestly but remained robust in the fully adjusted Model 2, in which each 1-point increment was associated with an approximately 4% lower hazard (HR 0.956, 95% CI 0.947–0.965; *P* < 0.001; Table 2).

**Table 2:** Association between DI-GM and risk of incident GERD.

Characteristics	Case/total (%)	GERD			
		Model 1		Model 2	
		HR (95% CI)	*P* value	HR (95% CI)	*P* value
DI-GM	12,271/133,915 (9.2)	0.942 (0.933–0.951)	< 0.001	0.956 (0.947–0.965)	< 0.001
DI-GM group					
0–3	2,882/28,495 (10.1)	Ref		Ref	
4	2,434/25,024 (9.7)	0.928 (0.879–0.979)	0.006	0.953 (0.903–1.006)	0.080
5	2,479/26,899 (9.2)	0.863 (0.817–0.910)	< 0.001	0.899 (0.852–0.949)	< 0.001
≥6	4,476/53,497 (8.4)	0.758 (0.723–0.794)	< 0.001	0.812 (0.774–0.851)	< 0.001
*P* for trend			< 0.001		< 0.001
Beneficial to gut microbiota	12,271/133,915 (9.2)	0.930 (0.920–0.940)	< 0.001	0.947 (0.936–0.957)	< 0.001
Unfavorable to gut microbiota	12,271/133,915 (9.2)	0.972 (0.952–0.992)	0.006	0.979 (0.959–0.999)	0.039

Model 1 was adjusted for age and sex. Model 2 was based on Model 1 and further adjusted for ethnicity, Townsend deprivation index, education, smoking status, alcohol status, MVPA, CVD, diabetes, hypertension, anxiety, depression, and sleep disorder. DI-GM, dietary index for gut microbiota; GERD, gastroesophageal reflux disease; MVPA, moderate-to-vigorous physical activity; CVD, cardiovascular disease.

A clear dose–response pattern was evident when DI-GM was modeled categorically. Relative to the lowest group (0–3), the fully adjusted HRs were 0.953 (95% CI 0.903–1.006; *P* = 0.080) for DI-GM = 4, 0.899 (95% CI 0.852–0.949; *P* < 0.001) for DI-GM = 5, and 0.812 (95% CI 0.774–0.851; *P* < 0.001) for DI-GM ≥6, with a significant linear trend (*P* for trend < 0.001). Decomposition showed that the inverse association was more pronounced for the beneficial sub-score (per 1-point increment HR 0.947, 95% CI 0.936–0.957; *P* < 0.001), whereas the unfavorable sub-score showed only a weak association (per 1-point increment HR 0.979, 95% CI 0.959–0.999; *P* = 0.039; Table 2). Because the unfavorable sub-score has a narrow integer range of 0–4, we did not rely on restricted cubic spline modeling for this sub-score in the main interpretation.

**Figure 2:**
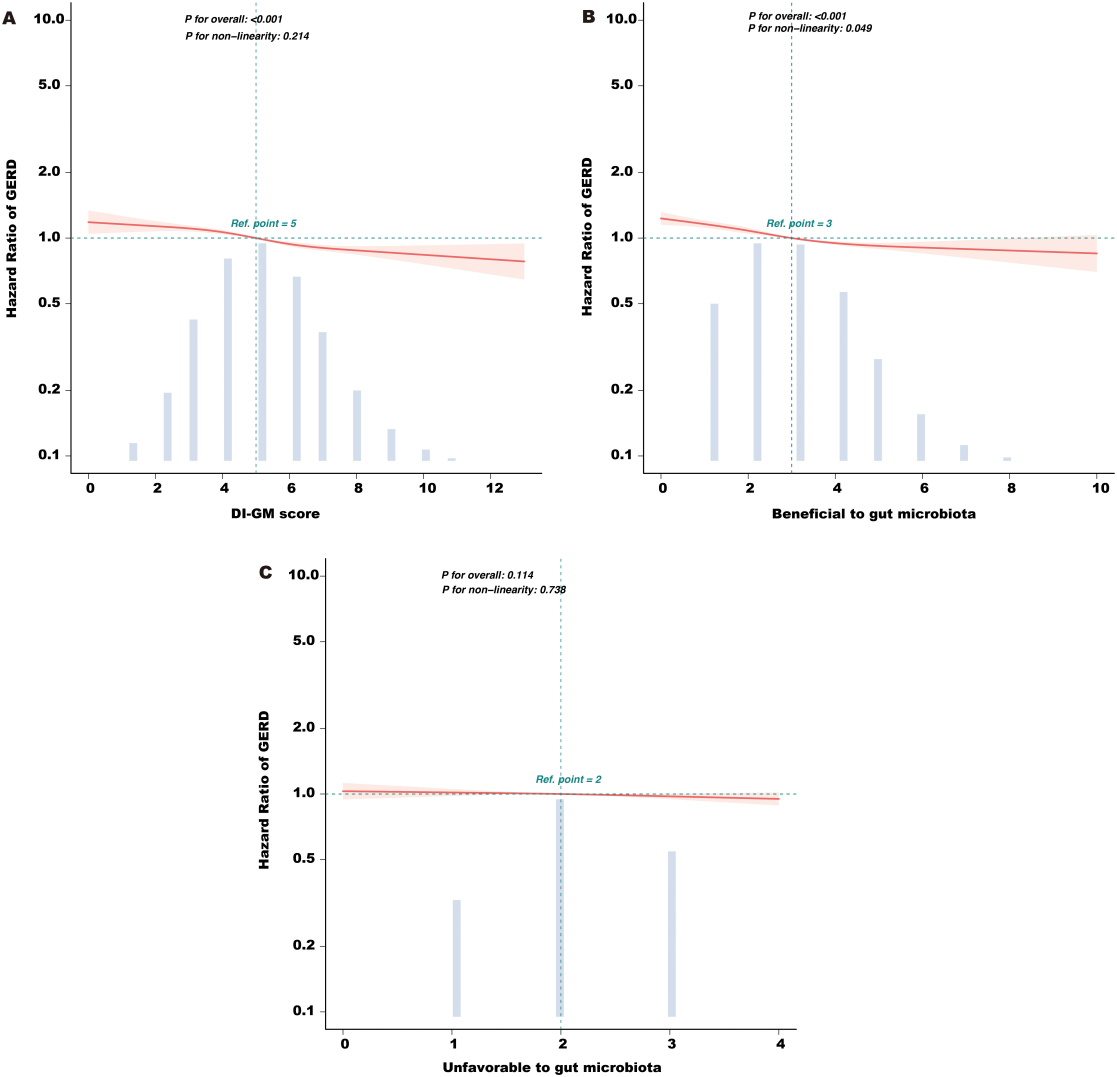
Restricted cubic spline analyses of the associations between DI-GM-related scores and the risk of incident GERD. **(A)** DI-GM score; **(B)** beneficial component score; **(C)** unfavorable component score. Hazard ratios are shown with 95% confidence intervals. Reference points were set at 5, 3, and 2, respectively. The model was adjusted for age, sex, ethnicity, Townsend deprivation index, education, smoking status, alcohol status, MVPA, CVD, diabetes, hypertension, anxiety, depression, and sleep disorder. DI-GM, dietary index for gut microbiota; GERD, gastroesophageal reflux disease; MVPA, moderate-to-vigorous physical activity; CVD, cardiovascular disease.

Restricted cubic spline analyses showed an overall inverse association between the total DI-GM score and incident GERD, with no significant non-linearity (*P* for overall < 0.001; *P* for non-linearity = 0.214; Figure 2A). A similar inverse association was observed for the beneficial sub-score, with mild non-linearity (*P* for overall < 0.001; *P* for non-linearity = 0.049; Figure 2B). For the unfavorable sub-score, given its small number of distinct integer values, the linear Cox estimate (HR 0.979; *P* = 0.039) was taken as the primary dose–response inference, with the restricted cubic spline (*P* for overall = 0.114; *P* for non-linearity = 0.738; Figure 2C) regarded as exploratory; together these indicate at most a weak inverse association. Kaplan–Meier analysis showed progressively lower cumulative incidence of GERD across higher DI-GM groups, with curves separating early in follow-up and continuing to diverge thereafter (log-rank *P* < 0.001; Supplementary Figure 2).

### 3.3 Subgroup analyses

The inverse association between DI-GM and incident GERD was largely consistent across subgroups defined by age, sex, smoking, alcohol, MVPA, and prevalent CVD, diabetes, hypertension, anxiety, depression, and sleep disorder (Supplementary Figure 3). After correction for multiple testing, no significant interactions were identified (all *P* for interaction > 0.05), supporting the robustness and generalizability of the association across major demographic and clinical subgroups.

### 3.4 Component-level contributions of DI-GM to GERD risk

**Figure 3:**
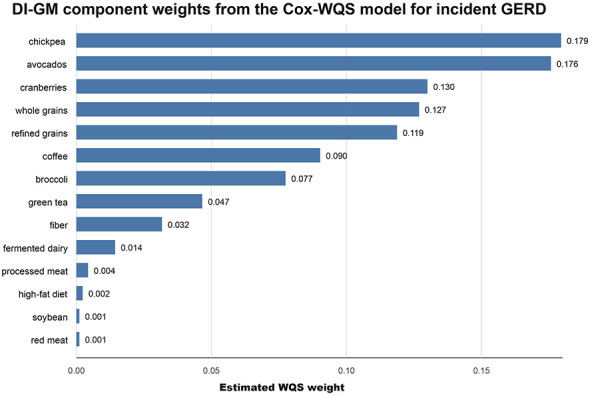
Component weights of DI-GM in the Cox weighted quantile sum regression model for incident GERD. The Cox-WQS model was adjusted for age, sex, ethnicity, Townsend deprivation index, education, smoking status, alcohol status, MVPA, CVD, diabetes, hypertension, anxiety, depression, and sleep disorder. Higher DI-GM component scores indicate a healthier dietary pattern; all components were coded in the same protective direction before WQS modeling, so for the unfavorable components (including refined grains) a higher score indicates lower or no intake. The x-axis shows the estimated WQS weight of each component; these weights, which sum to 1, reflect each component's contribution to the protective association in this consistent direction. DI-GM, dietary index for gut microbiota; GERD, gastroesophageal reflux disease; MVPA, moderate-to-vigorous physical activity; CVD, cardiovascular disease.

To assess the relative contribution of individual DI-GM components, a Cox-WQS regression model was fitted with weights constrained to sum to one (Figure 3). In the fully adjusted model, the composite WQS index was significantly inversely associated with incident GERD, indicating that joint variation across DI-GM components is associated with lower GERD risk. The component weights indicated that this association was concentrated in a few key contributors rather than shared equally. Chickpeas received the highest weight (0.179), followed by avocado (0.176), cranberries (0.130), whole grains (0.127), and refined grains (0.119); together these five components contributed approximately 73.1% of the total weight. Coffee (0.090), broccoli (0.077), and green tea (0.047) made moderate contributions, whereas dietary fiber, fermented dairy, processed meat, high-fat diet, soybeans, and red meat received relatively low weights. Overall, the WQS analysis indicated that the inverse association of DI-GM with GERD was driven mainly by chickpeas, avocado, cranberries, whole grains, and lower refined-grain intake.

### 3.5 Mediation by BMI and phenotypic age acceleration

**Figure 4:**
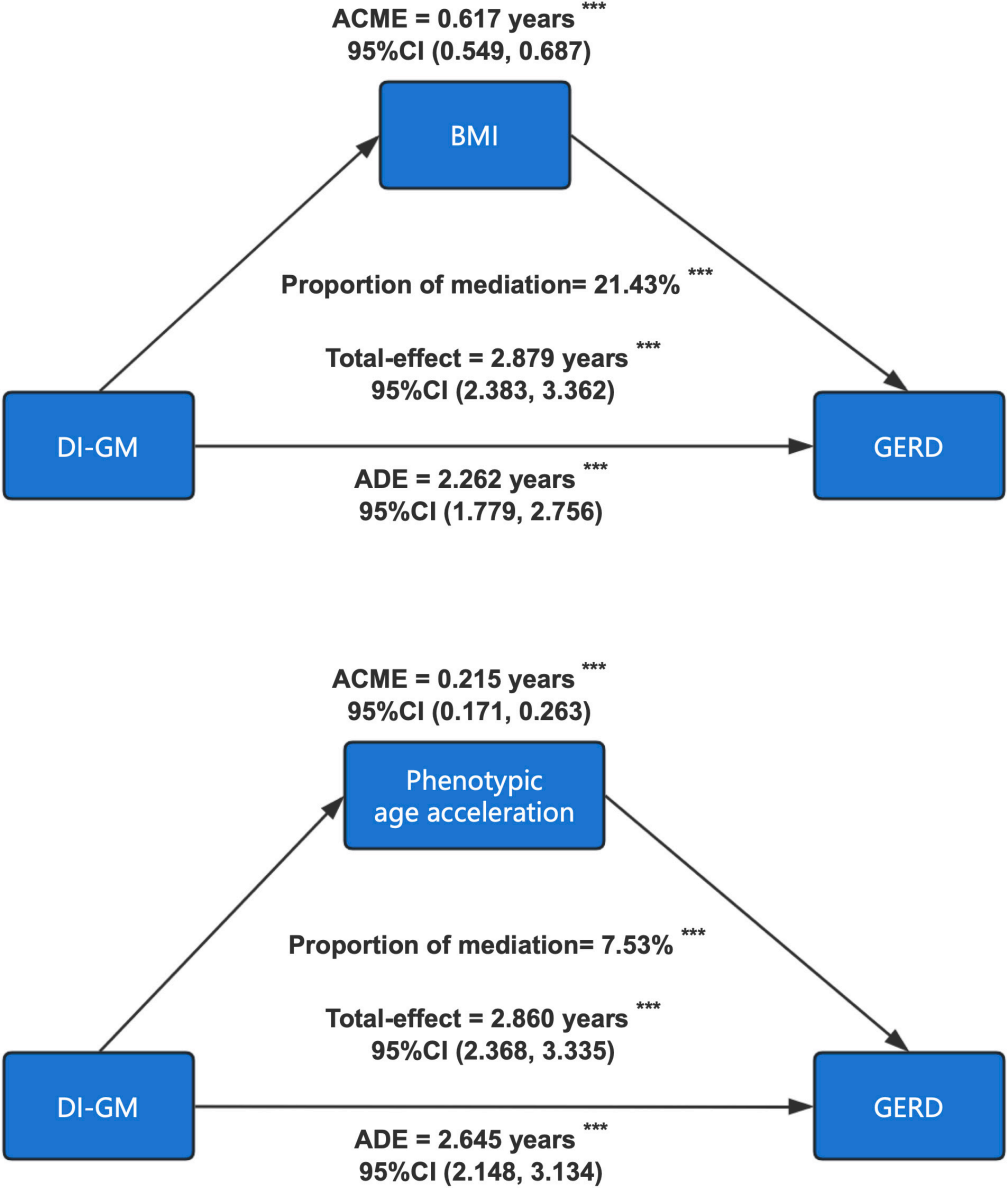
Exploratory mediation analysis of phenotypic age acceleration and BMI in the association between DI-GM and GERD. Phenotypic age acceleration was defined as the residual from regressing phenotypic age on chronological age. Models were adjusted for age, sex, ethnicity, Townsend deprivation index, education, smoking status, alcohol status, and MVPA. ACME, ADE and total effect are expressed as differences in model-predicted time to GERD (in years) for a one-point increase in DI-GM and are not hazard ratios; the proportion of mediation is unitless and was calculated as ACME divided by the total effect from the unrounded estimates (BMI, 21.43%; phenotypic age acceleration, 7.53%). As the estimates derive from a parametric accelerated failure time (AFT) model, this predicted-time scale is model-based and can exceed the observed follow-up. *** indicates *P* < 0.001. DI-GM, dietary index for gut microbiota; GERD, gastroesophageal reflux disease; BMI, body mass index; MVPA, moderate-to-vigorous physical activity; AFT, accelerated failure time; ACME, average causal mediation effect; ADE, average direct effect.

Given the established roles of adiposity and biological aging in upper gastrointestinal disease, we examined whether BMI and phenotypic age acceleration mediated the DI-GM–GERD association (Figure 4; Supplementary Table 4). Because these analyses used an accelerated failure time outcome model, the total effect, ACME and ADE are expressed as differences in model-predicted time (in years) to incident GERD, with positive values indicating a protective (delaying) association. Both BMI and phenotypic age acceleration were significant mediators (both *P* < 0.001). The association of DI-GM with each mediator was in the expected direction: each 1-point increase in DI-GM was associated with a lower phenotypic age acceleration (β = −0.222, 95% CI −0.235 to −0.208; *P* < 0.001; Supplementary Table 4) and a lower BMI (β = −0.281, 95% CI −0.294 to −0.269; *P* < 0.001). BMI mediated 21.43% of the total association (ACME 0.617 years, 95% CI 0.549–0.687; total effect 2.879, 95% CI 2.383–3.362), and phenotypic age acceleration mediated 7.53% (ACME 0.215, 95% CI 0.171–0.263; total effect 2.860, 95% CI 2.368–3.335). A substantial direct effect persisted after accounting for each mediator (BMI model ADE 2.262, 95% CI 1.779–2.756; phenotypic age acceleration model ADE 2.645, 95% CI 2.148–3.134; both *P* < 0.001).

### 3.6 Plasma proteomic signatures linking DI-GM to GERD

**Figure 5:**
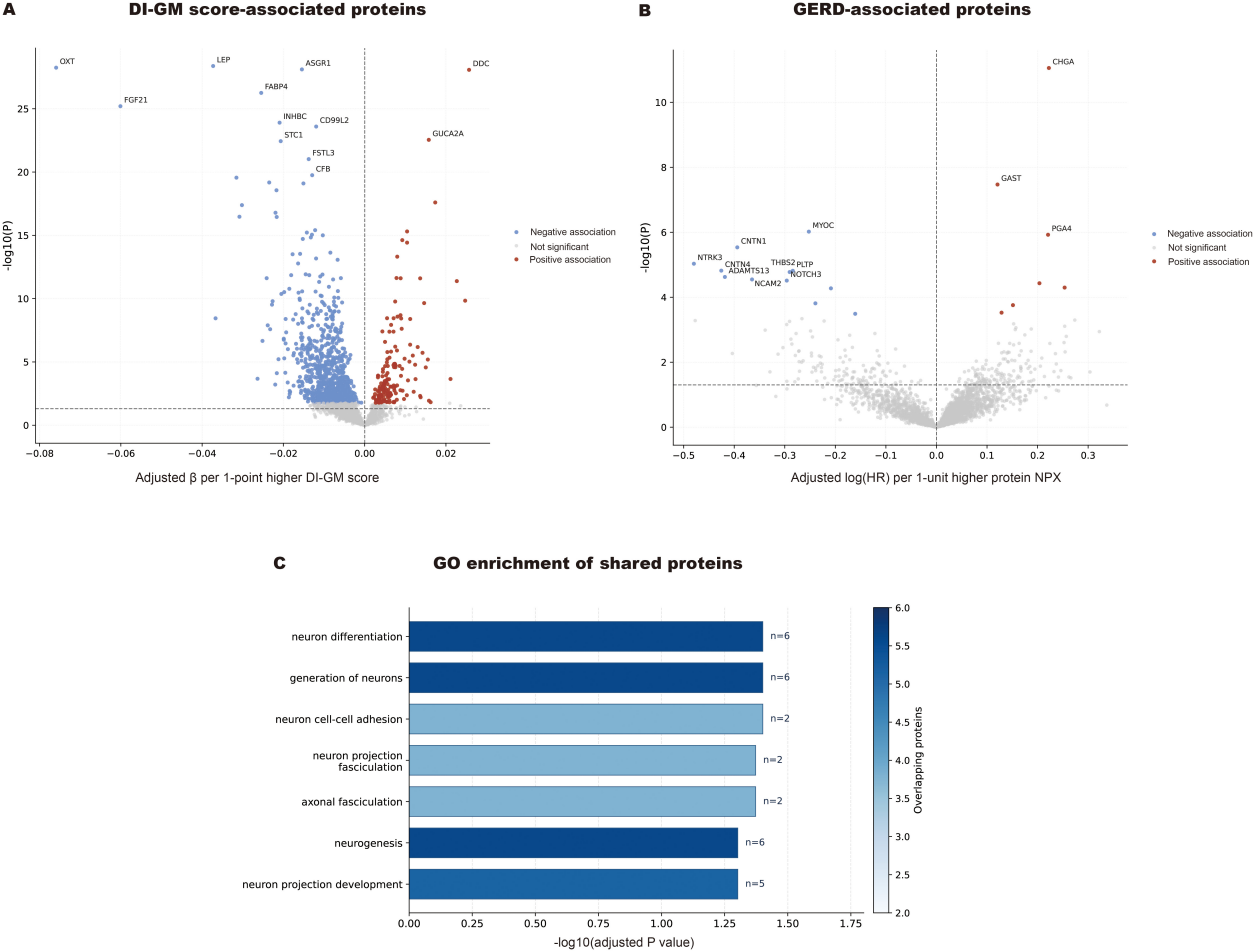
Proteomic signatures associated with the DI-GM score and GERD, and GO enrichment of shared proteins. **(A)** Volcano plot showing associations between the DI-GM score and plasma proteins. **(B)** Volcano plot showing associations between plasma proteins and incident GERD. **(C)** GO biological-process enrichment of the nine shared proteins positively associated with the DI-GM score and inversely associated with incident GERD. Models were adjusted for age, sex, ethnicity, Townsend deprivation index, education, smoking status, alcohol status, MVPA, CVD, diabetes, hypertension, anxiety, depression, and sleep disorder. GO:BP enrichment significance was defined as a Benjamini–Hochberg-adjusted *P* < 0.05. DI-GM, dietary index for gut microbiota; GERD, gastroesophageal reflux disease; GO, Gene Ontology

To explore potential molecular intermediates, we examined the associations of DI-GM with plasma proteins, and of plasma proteins with incident GERD, in 15,506 participants with baseline Olink proteomic data (2,910 proteins). After Bonferroni correction, 312 plasma proteins were significantly associated with the DI-GM score (Figure 5A; Supplementary Table 5). The most strongly associated proteins included leptin (LEP; β = −0.037, 95% CI −0.044 to −0.031; Bonferroni-adjusted *P* = 1.22 × 10^−25^), oxytocin (OXT; β = −0.076, 95% CI −0.089 to −0.063), asialoglycoprotein receptor 1 (ASGR1), fatty acid-binding protein 4 (FABP4), and fibroblast growth factor 21 (FGF21), all inversely associated with DI-GM, whereas DOPA decarboxylase (DDC), guanylate cyclase activator 2A (GUCA2A), and NEL-like 1 (NELL1) were positively associated with DI-GM. Cox models identified 19 plasma proteins associated with incident GERD at FDR < 0.05 (Figure 5B; Supplementary Table 6); higher levels of chromogranin A (CHGA; HR 1.249, 95% CI 1.172–1.331; *P* = 8.77 × 10^−25^), gastrin (GAST; HR 1.128, 95% CI 1.081–1.178), pepsinogen A4 (PGA4), LEP, and lipopolysaccharide-binding protein (LBP) were associated with higher GERD risk.

Cross-referencing the two sets (FDR < 0.05 in both) identified 13 shared proteins (Supplementary Table 7). The nine proteins positively associated with DI-GM (MYOC, CNTN1, NTRK3, CNTN4, PLTP, ADAMTS13, NCAM2, NOTCH3, and ADAMTS8) were uniformly associated with lower GERD risk; among proteins inversely associated with DI-GM, LEP, LBP, and TNFRSF4 were associated with higher GERD risk, whereas THBS2 was inversely associated with DI-GM but also with lower GERD risk. GO:BP enrichment analysis of the nine shared proteins positively associated with DI-GM and inversely associated with GERD showed over-representation of neurobiological processes, including neuron generation, neuron differentiation, neurogenesis, neuron projection development, neuron cell–cell adhesion, axonal fasciculation, and neuron projection fasciculation (all Benjamini–Hochberg-adjusted *P* < 0.05; Figure 5C; Supplementary Table 8). Given the modest number of proteins involved, these enrichment results are exploratory and hypothesis-generating.

### 3.7 Sensitivity analyses

The inverse association between DI-GM and incident GERD was robust across all sensitivity analyses. Restricting the analysis to participants with two or more dietary recalls (*n* = 82,340) yielded consistent estimates (per 1-point HR 0.966, 95% CI 0.954–0.977; ≥6 vs. 0–3 HR 0.847, 95% CI 0.795–0.902; Supplementary Table 9). Excluding incident GERD occurring within the first 2 years of follow-up gave similar results (per 1-point HR 0.958, 95% CI 0.949–0.968; ≥6 HR 0.822, 95% CI 0.782–0.865; Supplementary Table 10), as did re-anchoring follow-up to the date of the first completed WebQ (per 1-point HR 0.956, 95% CI 0.947–0.966; ≥6 HR 0.815, 95% CI 0.776–0.857; Supplementary Table 11) and additional adjustment for total energy intake (per 1-point HR 0.956; Supplementary Table 12). Although the global proportional hazards test indicated departures attributable to prevalent depression and sleep disorder (χ^2^ = 34.675, df = 17, *P* = 0.007), stratifying by these two covariates restored the global assumption (χ^2^ = 14.329, df = 15, *P* = 0.501) and left the DI-GM estimates essentially unchanged (per 1-point HR 0.956, 95% CI 0.947–0.965; ≥6 HR 0.811, 95% CI 0.774–0.851; Supplementary Table 13). Accounting for the competing risk of death using the Fine–Gray model yielded consistent subdistribution hazard ratios (per 1-point sHR 0.957, 95% CI 0.948–0.966; ≥6 sHR 0.817, 95% CI 0.779–0.857; Supplementary Table 14). For the highest vs. lowest DI-GM category, the E-value was 1.77 for the point estimate and 1.63 for the confidence-interval limit nearer the null, indicating that an unmeasured confounder would need a moderately strong association with both exposure and outcome to fully explain the observed association.

The proteomic findings were similarly robust. Complete-case analyses without imputation closely reproduced the imputed-data results for both the DI-GM–protein (r = 0.997) and protein–GERD (r = 0.999) associations (Supplementary Table 15) and retained all shared proteins (Supplementary Table 16). The proteomic subsample was broadly representative of the full analytic cohort (standardized mean differences ≤ 0.02 for age, sex, BMI, and incident GERD; Supplementary Table 17). The 13 shared proteins significantly exceeded the number expected by chance (expected 6.9; ratio 1.88; hypergeometric *P* = 0.0044), and the GO:BP neurobiological enrichment was reproduced when the analysis was restricted to the nine directionally concordant proteins but not when undirected or directionally discordant protein sets were used (Supplementary Table 18).

## 4 Discussion

In this large prospective cohort from the UK Biobank, we found that a higher DI-GM score was associated with a significantly lower risk of incident GERD, with a clear dose–response relationship. The association was robust across multiple covariate adjustment models and consistent across major demographic and clinical subgroups. Exploratory mediation analyses indicated that BMI and phenotypic age acceleration partially mediated the association, while a direct association between DI-GM and GERD persisted after accounting for these mediators. Plasma proteomic analyses further identified 13 shared proteins linking DI-GM and GERD with largely consistent directionality. To our knowledge, this is the first study to systematically evaluate the DI-GM–GERD relationship in a large population-based cohort and to integrate epidemiological, biological-aging, mediation, and proteomic analyses within a single analytical framework. We emphasize at the outset, however, that the gut microbiome was not measured; the interpretations that follow, including those linking DI-GM to the microbiota or to downstream proteins, are therefore inferential and hypothesis-generating.

Previous studies have shown that specific dietary patterns such as the Mediterranean diet, DASH diet, and fiber-rich diets are associated with lower GERD risk, while Western dietary patterns, high-fat intake, and processed food consumption are associated with aggravated GERD symptoms (23–25). However, most prior work has focused on isolated foods or nutrients and could not fully capture the integrative effect of diet on the gut microbiota. As a recently proposed composite indicator, DI-GM integrates 14 evidence-based dietary components and serves as a feasible population-level proxy for diet–microbiota relationships. Since its introduction, higher DI-GM scores have been associated with lower risks of obesity, type 2 diabetes, metabolic syndrome, depression, and colorectal cancer, suggesting its potential relevance to chronic disease risk across multiple organ systems (26–29). The present study extends DI-GM to GERD, an upper gastrointestinal outcome, and shows that each 1-point increase in DI-GM is associated with an approximately 4% lower risk of incident GERD (HR 0.956, 95% CI 0.947–0.965), with a graded reduction across DI-GM groups (≥6 vs. 0–3: HR 0.812, 0.774–0.851). This inverse association is directionally consistent with those reports, supporting the relevance of the overall dietary pattern, rather than individual foods, to GERD risk. RCS analyses showed a modest inverse association between DI-GM and GERD risk, with the most apparent risk reduction confined to higher DI-GM scores. The inverse association with GERD appeared more pronounced for the beneficial sub-score (HR 0.947, 95% CI 0.936–0.957). In contrast, the unfavorable sub-score showed only weak evidence of association in the linear Cox model (HR 0.979, 95% CI 0.959–0.999), and its narrow discrete range limited meaningful spline-based dose–response assessment. The conclusion that DI-GM was mainly driven by beneficial components should therefore be interpreted cautiously and rests on the stronger and more consistent association observed for the beneficial sub-score, rather than on a direct statistical contrast between the two RCS curves.

WQS analyses indicated that the component-level association was concentrated mainly in chickpeas (0.179), avocados (0.176), cranberries (0.130), whole grains (0.127) and lower refined-grain intake (0.119), which together accounted for approximately 73% of the total weight, rather than being distributed equally across components. We treat these weights as exploratory relative-contribution estimates that indicate which components contributed most to the observed cohort-level association, rather than as a basis for component-specific dietary prescriptions, given the observational design, the unvalidated nature of the exposure, and the absence of direct microbiome measurement (30). Accordingly, the WQS analysis was used to identify the components contributing most to the statistical association between DI-GM and incident GERD, not to generate universal clinical recommendations; dietary advice for GERD should remain individualized, particularly because some DI-GM components may provoke symptoms in susceptible individuals. A further consideration concerns the apparent tension between our findings and the clinical dietary-trigger literature. Several components rated favorably by the DI-GM (including coffee, tea, acidic foods such as cranberries, and gas-producing legumes such as chickpeas) are recognized in the American College of Gastroenterology (ACG) guideline and elsewhere as potential triggers of reflux symptoms (1). This tension is largely reconciled by distinguishing two questions that operate on different timescales: the short-term provocation of heartburn in individuals with established GERD, and the long-term incidence of new-onset disease examined here over a median of 13.6 years. A food that transiently provokes symptoms in a sensitized individual may, through sustained effects on the gut microbiota, adiposity, and systemic inflammation, nonetheless be associated with lower long-term disease incidence at the population level. Moreover, most of the foods cited as triggers contributed little to the protective signal in our WQS analysis (high-fat pattern 0.002, soybeans 0.001, green tea 0.047, coffee 0.090), whereas the dominant contributors were chickpeas, avocados, cranberries, and whole grains; of these, only chickpeas and cranberries also appear on trigger lists, and their long-term, population-level association is distinct from any acute symptom-provoking potential in sensitized individuals. Consistent with current ACG guideline, which favors individualized avoidance of demonstrably symptomatic foods rather than blanket restriction, our population-level findings on incident disease are complementary to, rather than in conflict with, individual-level symptom management. Accordingly, we do not recommend microbiota-favorable foods indiscriminately for individuals with active reflux symptoms.

Exploratory mediation analyses showed that BMI and phenotypic age acceleration each significantly mediated the DI-GM–GERD association, with the proportion mediated by BMI (21.43%) larger than that of phenotypic age acceleration (7.53%). The mediating role of BMI is biologically plausible: adiposity increases intra-abdominal pressure, promotes hiatal hernia formation, lowers lower gastroesophageal sphincter tone, and amplifies low-grade systemic inflammation, all established risk factors for GERD, and higher DI-GM scores are closely linked to lower BMI (31, 32). The smaller mediation by phenotypic age acceleration suggests that microbiota-favorable dietary patterns may relate to lower GERD risk partly through systemic inflammation, metabolic homeostasis, and the cumulative burden of organ-system aging (33). As these proportions were estimated in separate single-mediator models, they should not be summed; in each, a direct association between DI-GM and GERD remained after accounting for the mediator, indicating that additional pathways remain to be identified. Plasma proteomic analyses provided further clues to this residual direct association.

Among the 13 shared proteins, directional consistency was high. Among proteins inversely associated with DI-GM and positively associated with GERD risk, LEP, LBP, and TNFRSF4 showed concordant directionality, with higher DI-GM corresponding to lower protein levels and lower protein levels to lower GERD risk; these reflect adiposity-related inflammation, lipopolysaccharide-driven endotoxemia, and T-cell co-stimulatory signaling, respectively, consistent with established roles of chronic low-grade inflammation in reflux-related gastroesophageal injury (34–36). Because these proteins are closely tied to adiposity, this component of the shared-protein signal may largely reflect the BMI-related (adiposity–inflammation) pathway identified in our mediation analysis rather than an independent mechanism. On the protective side, a small number of neurobiology-related proteins were also identified, including the contactins (CNTN1, CNTN4), NTRK3, NCAM2, and NOTCH3, and GO enrichment of the directionally concordant proteins highlighted neuron generation, neuron differentiation, and axon-fasciculation terms. We interpret this neurodevelopment signal cautiously: it is supported by relatively few proteins, none of the intermediate steps that might link it to reflux (including the gut microbiome, short-chain fatty acids, vagal or enteric nervous system function, and gastroesophageal motility) was measured, and we did not test mediation through these proteins. Three of these terms (neuron cell–cell adhesion, axonal fasciculation, and neuron projection fasciculation) were each supported by only two proteins at a Benjamini–Hochberg-adjusted P of approximately 0.04, and the enriched terms are driven largely by proteins carrying neuronal adhesion or signaling annotations (CNTN1, CNTN4, NCAM2, NTRK3, NOTCH3); they may therefore reflect the shared annotation of these molecules rather than a GERD-specific pathway. It should therefore be regarded as a speculative, hypothesis-generating observation requiring mechanistic validation, rather than as evidence of a microbiota–neural pathway protecting gastroesophageal function.

These findings are associational and should be interpreted cautiously. The inverse association between DI-GM and incident GERD was modest in absolute terms, with an absolute risk difference of approximately 1.7% points between the highest and lowest groups, and was statistically detectable mainly at the highest DI-GM categories. Because higher DI-GM was accompanied by a broader health-conscious and socioeconomically advantaged profile, residual confounding by overall diet quality and lifestyle is an important alternative explanation, and the gut microbiome was not measured. We therefore do not propose DI-GM as a stand-alone screening tool, do not claim benefit across the full score range, and do not derive component-specific clinical prescriptions from the WQS weights. Causal and interventional evidence, together with external validation and direct microbiome assessment, would be required before any public-health or clinical application could be considered.

The strengths of the present study include analyses in the large, prospective, and deeply phenotyped UK Biobank cohort; the combination of traditional epidemiological methods (Cox regression, RCS, Kaplan–Meier, subgroup analyses) with novel methodologies (Cox-WQS regression, counterfactual mediation); and the integration of plasma proteomic data to provide a multi-level evidence chain from dietary pattern to candidate molecular pathway. A further strength is the comprehensive set of sensitivity analyses used to probe the robustness of the findings, including restriction to participants with two or more 24-h recalls, exclusion of the first 2 years of follow-up, re-anchoring of follow-up to the dietary-assessment date, additional adjustment for total energy intake, a Fine–Gray model accounting for competing mortality risk, and E-values quantifying robustness to unmeasured confounding. Across all of these, the inverse association between DI-GM and incident GERD was essentially unchanged, indicating that the principal findings are robust to exposure misclassification, reverse causation, competing risk, and plausible unmeasured confounding. Several limitations should also be acknowledged. First, the gut microbiome was not measured; DI-GM is a dietary index hypothesized to reflect microbiota-favorable intake, so the diet–microbiota link is inferential and we do not claim a tested microbiota-mediated mechanism. Second, diet was assessed by the Oxford WebQ 24-h recall, with 38.5% of participants completing a single recall; this short-term, median-dichotomized measure is subject to non-differential misclassification that would generally bias the association toward the null, so our estimates are likely conservative, although a sensitivity analysis restricted to participants with two or more recalls was consistent. Because the entire DI-GM exposure is derived from this single self-reported instrument, residual exposure misclassification cannot be excluded. Third, higher DI-GM tracked a gradient of greater socioeconomic advantage and a more health-conscious profile, and UK Biobank participants (predominantly white, and further selected by WebQ completion) are healthier than the general population; despite extensive adjustment, residual confounding by overall diet quality and lifestyle cannot be excluded, and external validation in more diverse cohorts is required. Fourth, GERD was ascertained from ICD-10 K21 and self-report and thus represents medically attended GERD, which under-records community-managed reflux and may be detected differentially by healthcare contact in a direction that cannot be predetermined, since this contact correlates with diet quality; baseline proton pump inhibitor or H2-receptor antagonist use, a recognized microbiota modifier, was not adjusted for, although an E-value analysis (1.77 for the point estimate, 1.63 for the upper confidence limit; ≥6 vs. 0–3) suggested reasonable robustness (37). Confirmation in analyses incorporating prescription-based or uniformly ascertained outcomes (independent of healthcare-seeking) would help establish whether the association extends to community-managed reflux. Reverse causation is also possible: because the K21 endpoint captures only coded, medically attended GERD, individuals with early or undiagnosed reflux may have modified their diet before a formal diagnosis (for example, by reducing high-fat foods, coffee, alcohol, or citrus), which could lower DI-GM and inflate the apparent inverse association. The early separation of the cumulative-incidence curves is consistent with this possibility as well as with a true association. A lag analysis excluding the first 2 years of follow-up was essentially unchanged, providing some reassurance, but reverse causation operating over longer intervals cannot be excluded. Finally, the mediation and proteomic analyses were exploratory and hypothesis-generating: DI-GM and the mediators (BMI, phenotypic age acceleration) were measured at the same visit, precluding temporal ordering, and BMI is a shared cause of diet and GERD, so the proportions mediated should not be read as purely causal, while the proteomic subsample (11.6% of the cohort) had reduced power; these findings require validation in mechanistic, animal, and interventional studies.

## 5 Conclusion

In summary, in this large prospective UK Biobank cohort, higher DI-GM scores were associated with a significantly lower risk of incident, medically attended GERD (ICD-10 K21), with a graded dose–response relationship. Exploratory mediation analyses suggested that BMI and, to a lesser extent, phenotypic age acceleration may partly account for this association, and exploratory proteomic analyses identified candidate protein correlates, including inflammatory and metabolic proteins and, tentatively, neurodevelopment- and cell-adhesion-related proteins. These findings describe associations rather than established causal mechanisms; they provide epidemiological and hypothesis-generating molecular evidence on a microbiota-relevant dietary pattern and GERD, and warrant confirmation in mechanistic and interventional studies.

## Data Availability

The datasets presented in this article are not readily available because The UK Biobank data used in this study are available to bona fide researchers through application at https://www.ukbiobank.ac.uk. The present analyses were performed under UK Biobank application number 1099827. Requests to access the datasets should be directed to UK Biobank Access Management System: https://www.ukbiobank.ac.uk/enable-your-research/apply-for-access.
