# Supplementary material for: Dietary index for gut microbiota and risk of incident gastroesophageal reflux disease: a prospective cohort analysis integrating plasma proteomics in the UK Biobank
Source: Front Nutr. 2026 Jul 7;13:1880631. doi: 10.3389/fnut.2026.1880631 (PMC13385169; doi:10.3389/fnut.2026.1880631)
Supplement: Supplementary file 1 [file Table_1.docx]

**Supplementary Table 1** **Components and scoring criteria of DI-GM**

| **Component ᵃ** | **Foods Component** | **Scoring** |
| --- | --- | --- |
| **Beneficial to gut microbiota** | | |
| Avocados | Avocados | For each component, a score of 1 if consumption at or above the  sex-specific median, else 0. |
| Broccoli | Broccoli |  |
| Chickpea | Chickpeas |  |
| Coffee | Coffee |  |
| Cranberries | Cranberries |  |
| Fermented dairy | Yogurt, cheese, kefir, sour cream, buttermilk |  |
| Fiber | Fiber |  |
| Green tea | Green tea |  |
| Soybean | Soy products—Soy milk, Tofu |  |
| Whole grains | Grains defined as whole grains, containing the entire grain kernel—the bran, germ, and endosperm |  |
| **Unfavorable to gut microbiota** | | |
| High-fat diet (% energy) | Not applicable | 0 if consumption at or above 40% energy from fat, else 1. |
| Processed meat | Frankfurters, sausages, corned beef, and luncheon meat made from beef, pork, or poultry | For each remaining component, a score of 0 if consumption at or  above the sex-specific median, else 1. |
| Red meat | Beef, veal, pork, lamb, and game meat; excludes organ meat and cured meat |  |
| Refined grains | Refined grains that do not contain all components of the entire grain kernel |  |

Abbreviations: DI-GM, dietary index for gut microbiota.

.

**Supplementary Table 2 UK Biobank field IDs and definitions of exposures, outcomes, and covariates**

| **Variables** | **Field IDs** | **Definitions of variables** |
| --- | --- | --- |
| **Outcomes** | | |
| Gastroesophageal reflux disease | K21 | ICD 10 code |
|  | 1138 | Data-Coding in the UK biobank |
| **Exposures** | | |
| Avocado intake | 104100 | "How many medium avocados did you have?" |
| Broccoli intake | 104140 | "How many servings of broccoli did you have?" |
| Coffee, caffeinated | 26081 | Estimated intake from past 24 hours. |
| Coffee, decaffeinated | 26082 | Estimated intake from past 24 hours. |
| Dried fruit | 26092 | Estimated intake from past 24 hours. |
| Pulses intake c | 104010 | Estimated intake from past 24 hours. |
| Full fat yogurt | 26096 | Estimated intake from past 24 hours. |
| Low fat yogurt | 26102 | Estimated intake from past 24 hours. |
| High fat cheese | 26099 | Estimated intake from past 24 hours. |
| Medium and low fat cheese | 26103 | Estimated intake from past 24 hours. |
| Englyst fiber | 26017 | Estimated intake from past 24 hours. |
| Green tea | 100420 | "How many cups/mugs of green tea did you drink yesterday?" |
| Soy milk | 26136 | Estimated intake from past 24 hours. |
| Tofu intake | 103270 | "How many servings of tofu/tempeh/TVP/soya mince did you have?" |
| Soya dessert intake | 102170 | "How many servings/individual pots of soya ice cream, soya yogurt, other soya dessert did you have?" |
| Wholemeal bread | 26074 | Estimated intake from past 24 hours. |
| Wholemeal pasta, brown rice and other wholegrains | 26114 | Estimated intake from past 24 hours. |
| Muesli | 26105 | Estimated intake from past 24 hours. |
| Bran cereal | 26076 | Estimated intake from past 24 hours. |
| Other cereal | 26079 | Estimated intake from past 24 hours. |
| Oat cereal (sugar) | 26078 | Estimated intake from past 24 hours. |
| Oat cereal | 26077 | Estimated intake from past 24 hours. |
| Fat | 26008 | Fat from overall diet. Estimated intake from past 24 hours. |
| Energy | 26002 | Energy from overall diet. Estimated intake from past 24 hours. |
| Processed meat | 26122 | Estimated intake from past 24 hours. |
| Beef | 26066 | Estimated intake from past 24 hours. |
| Pork | 26117 | Estimated intake from past 24 hours. |
| Lamb | 26100 | Estimated intake from past 24 hours. |
| White bread | 26073 | Estimated intake from past 24 hours. |
| Other bread | 26072 | Estimated intake from past 24 hours. |
| Biscuit cereal | 26075 | Estimated intake from past 24 hours. |
| Biscuits | 26068 | Estimated intake from past 24 hours. |
| Pizza | 26116 | Estimated intake from past 24 hours. |
| Other desserts and cakes and pastries | 26085 | Estimated intake from past 24 hours. |
| Savoury snacks | 26134 | Estimated intake from past 24 hours. |
| Samosa, pakora | 26128 | Estimated intake from past 24 hours. |
| Savoury crackers | 26083 | Estimated intake from past 24 hours. |
| Grain dishes | 26097 | Estimated intake from past 24 hours. |
| **Covariates** | | |
| Age at recruitment | 21022 | Age was treated as a continuous variable |
| Sex | 31 | Sex of participant |
| Ethnicity | 21000 | Ethnic background |
| Townsend deprivation index | 22189 | Townsend deprivation index calculated immediately prior to participant joining UK Biobank. Based on the preceding national census output areas. Each participant is assigned a score corresponding to the output area in which their postcode is located. Townsend deprivation index was treated as a continuous variable and missing value with the average fill. |
| Education | 6138 | Data-Coding in the UK biobank: “College or university degree”, “A level/AS levels or equivalent”, “O levels/GCSEs or equivalent”, “CSEs or equivalent”, “NVQ or HND or HNC or equivalent”, “Other professional qualifications”, “None of the above”, “Prefer not to answer”. We combined “College or university degree” into a group, “A level/AS levels or equivalent” and “O levels/GCSEs or equivalent” and “CSEs or equivalent” and “NVQ or HND or HNC or equivalent” and “Other professional qualifications” into a group, “None of the above” into a group. |
| Body mass index | 21001 | BMI value here is constructed from height and weight measured during the initial Assessment Centre visit. Value is not present if either of these readings were omitted. |
| Phenotypic age |  |  |
|  | 30600 | Albumin |
|  | 30700 | Creatinine |
|  | 30740 | Glucose |
|  | 30710 | C-reactive protein |
|  | 30180 | Lymphocyte percentage |
|  | 30040 | Mean corpuscular volume |
|  | 30070 | Red blood cell distribution width |
|  | 30610 | Alkaline phosphatase |
|  | 30000 | White blood cell / leukocyte count |
| Smoking status | 20116 | Classified as Never (0) if Current tobacco smoking = prefer not to say (-3) or no (0) AND Past tobacco smoking = just tried once or twice (3) or I have never smoked (4).  Classified as Previous (1) if Current tobacco smoking = no (0) AND Past tobacco smoking = smoked on most or all days (1) or smoked occasionally (2). |
| Alcohol status | 20117 | Alcohol status was based on self-reported drinking habits at baseline and classified as never, previous, or current. |
| Meets MVPA recommendation | 22035 | Indicates whether a person met the 2017 UK Physical activity guidelines of 150 minutes of moderate activity per week or 75 minutes of vigorous activity. |
| CVD | 131296, 131298, 131300, 131302, 131304, 131306, 131360, 131362, 131364, 131366, 131368, 131378 | ICD-10 codes I20–I25, I60–I64, I69 |
| Hypertension | 131286 | ICD-10 code I10 |
| Diabetes | 130706, 130708, 130710, 130712, 130714 | ICD-10 codes E10–E14 |
| Anxiety | 130904, 130906 | F40–F41 |
| Depression | 130894, 130896 | F32–F33 |
| Sleep disorder | 131060 | G47 |

**Supplementary Table 3 Missingness profile of plasma proteins**

| **Metric** | **Value** |
| --- | --- |
| Proteins measured on the Olink platform | 2,923 |
| Proteins excluded because missingness was >20% | 13 |
| Proteins retained for analysis | 2,910 |
| Median missing rate among retained proteins | 7.35% |
| Interquartile range of missing rates | 3.02%-17.77% |
| Maximum missing rate among retained proteins | 19.77% |
| Minimum non-missing observations per retained protein | 12,441 |
| Median non-missing observations per retained protein | 14,367 |
| Maximum non-missing observations per retained protein | 15,243 |

Proteins with >20% missing NPX values were excluded. The primary analysis used protein-specific mean imputation for remaining missing NPX values; protein-specific complete-case analyses were conducted as sensitivity analyses.


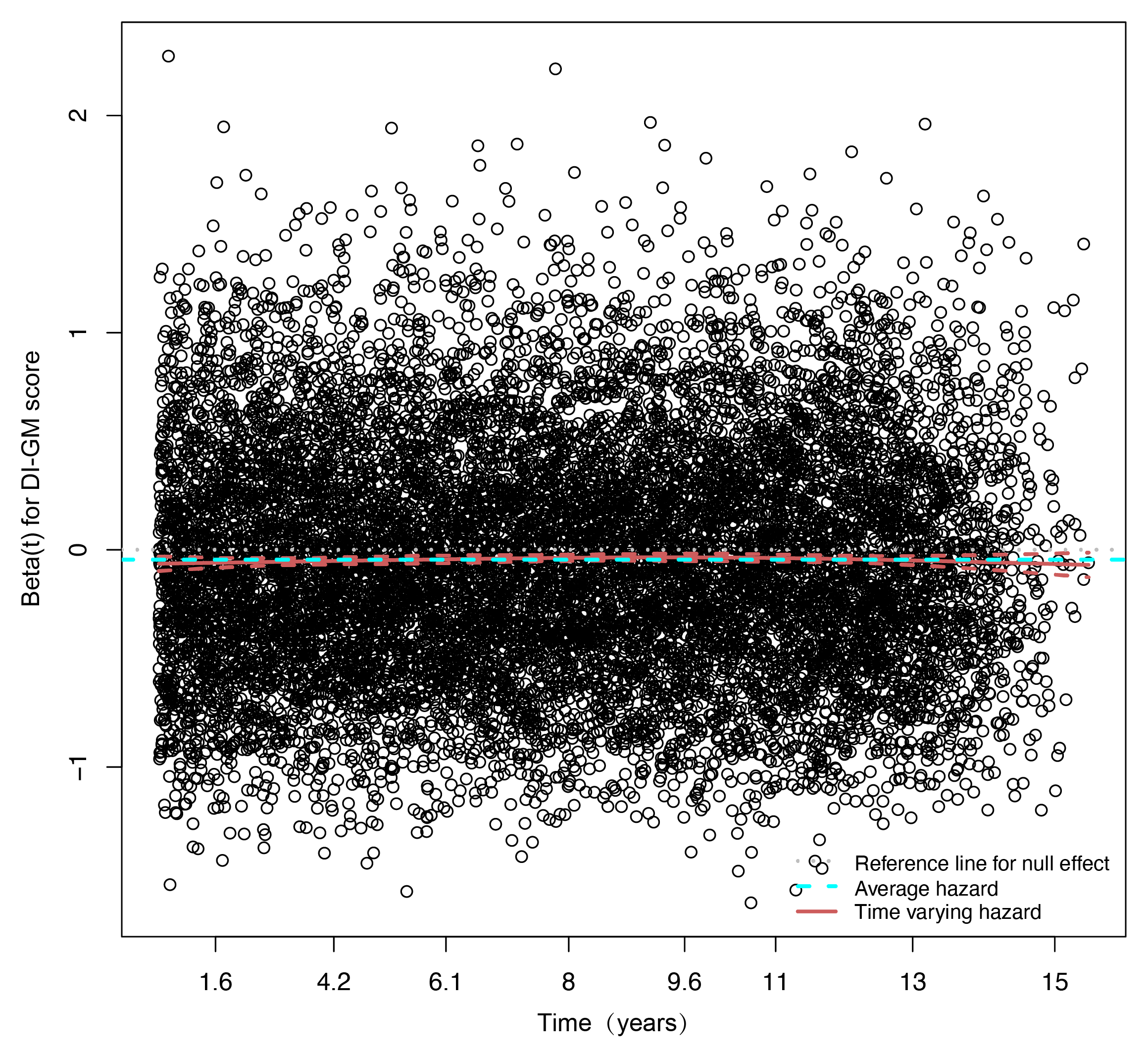


**Supplementary Figure 1 Scaled Schoenfeld residual plot for DI-GM in the fully adjusted Cox model (Model 2).**

Abbreviations: DI-GM, dietary index for gut microbiota.


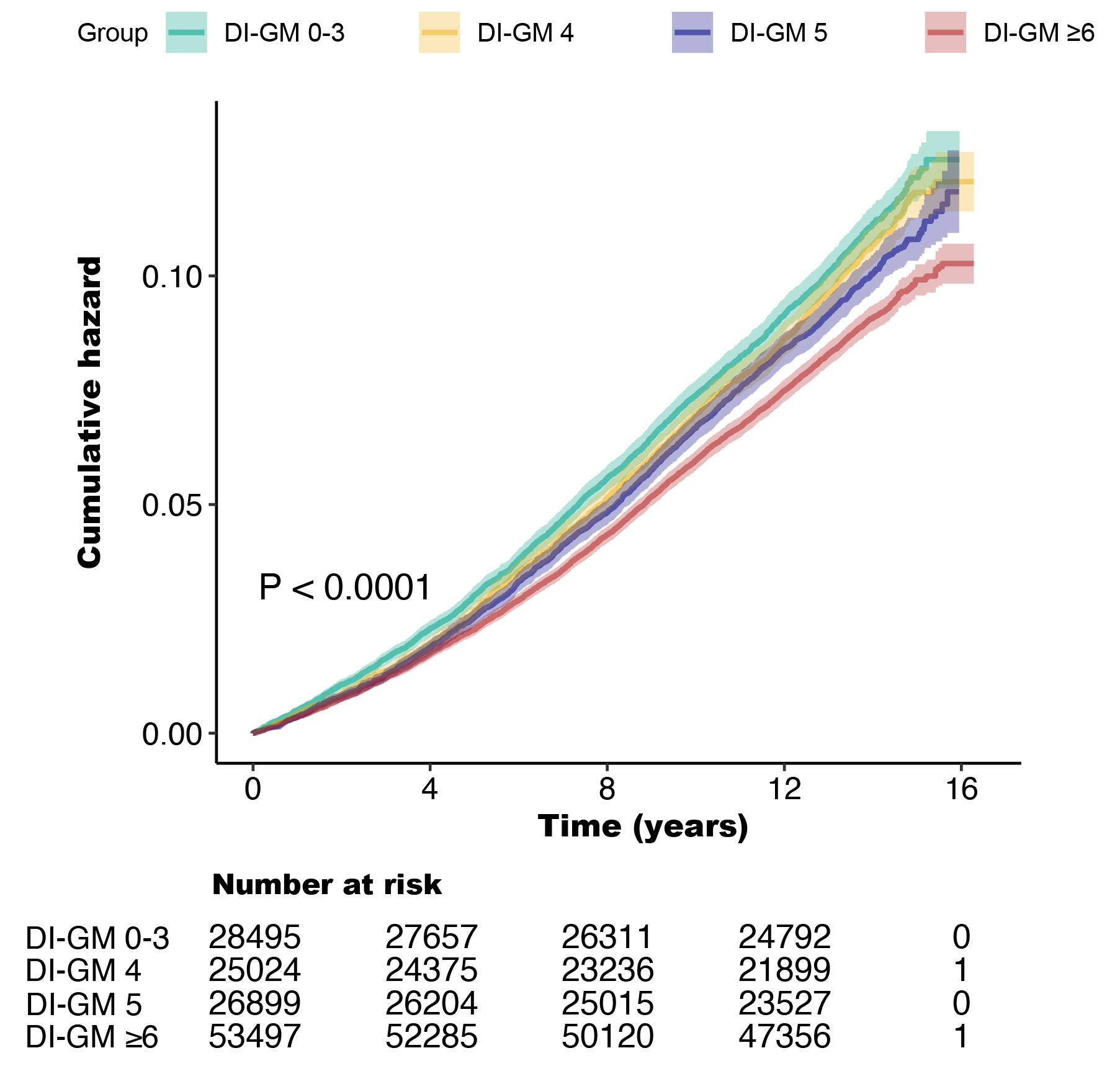


**Supplementary Figure 2 Kaplan–Meier curves for cumulative incidence of GERD by DI-GM group.**

Abbreviations: DI-GM, dietary index for gut microbiota; GERD, gastroesophageal reflux disease.


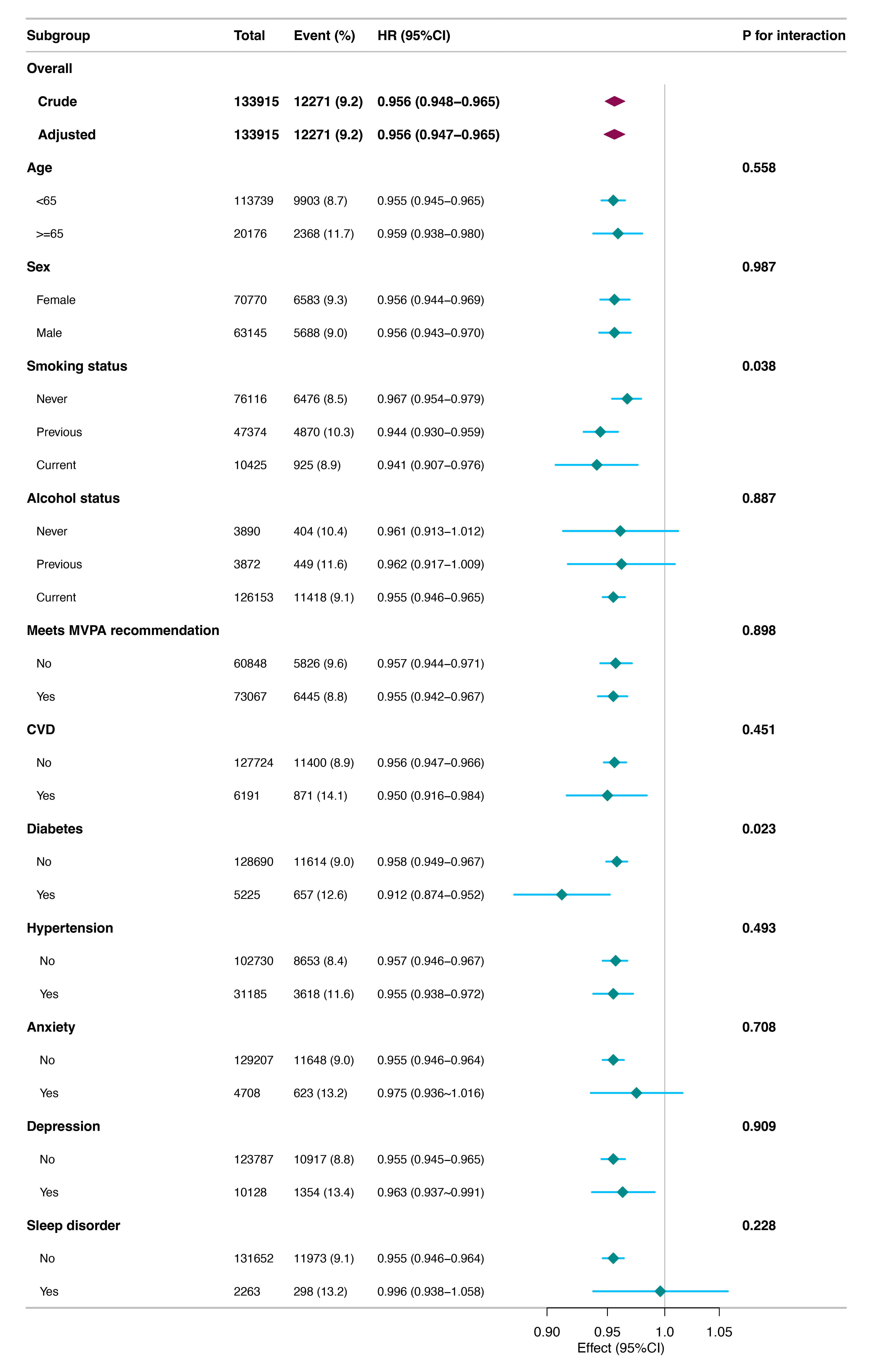


**Supplementary Figure 3 Subgroup analyses for the association between DI-GM score and risk of incident GERD.**

Note: Models were adjusted for age, sex, ethnicity, Townsend deprivation index, education, smoking status, alcohol status, MVPA, CVD, diabetes, hypertension, anxiety, depression, and sleep disorder, except for the stratifying variable.

Abbreviations: DI-GM, dietary index for gut microbiota; GERD, gastroesophageal reflux disease; MVPA, moderate-to-vigorous physical activity; CVD, cardiovascular disease.

**Supplementary Table 4 Path coefficients (a, b and c′) for the mediation of the association between DI-GM and incident GERD by body mass index and phenotypic age acceleration**

| **Path** | **Association** | **Model (scale)** | **β** | **95% CI** | ***P* value** |
| --- | --- | --- | --- | --- | --- |
| **Body mass index** | | | | | |
| *a* | DI-GM → BMI | Linear mediator model (kg/m²) | −0.281 | −0.294 to −0.269 | <0.001 |
| *b* | BMI → GERD | AFT outcome model (log-time) | −0.029 | −0.031 to −0.026 | <0.001 |
| *c′* | DI-GM → GERD (direct) | AFT outcome model (log-time) | 0.029 | 0.022 to 0.037 | <0.001 |
| **Phenotypic age acceleration** | | | | | |
| *a* | DI-GM → phenotypic age acceleration | Linear mediator model (years) | −0.222 | −0.235 to −0.208 | <0.001 |
| *b* | Phenotypic age acceleration → GERD | AFT outcome model (log-time) | −0.013 | −0.016 to −0.010 | <0.001 |
| *c′* | DI-GM → GERD (direct) | AFT outcome model (log-time) | 0.035 | 0.028 to 0.042 | <0.001 |

Note: Models were adjusted for age, sex, ethnicity, Townsend deprivation index, education, smoking status, alcohol status, and MVPA. All P values were below 0.001. The a-path is the coefficient from the linear mediator model (the mediator regressed on DI-GM and covariates), reported on the mediator's own scale (kg/m² for body mass index; years for phenotypic age acceleration). The b-path and direct effect (c′) are from the accelerated failure time (AFT) outcome model, reported on the log-time scale, on which a positive coefficient denotes a longer time to GERD onset. The corresponding average causal mediation effect, average direct effect and total effect on the predicted-time scale (in years), together with the proportion mediated, are presented in Figure 4. Because the predicted-time scale is a nonlinear transformation of the log-time scale, the proportion mediated computed from the log-time coefficients above approximates but does not exactly equal the value reported in Figure 4.

Abbreviations: AFT, accelerated failure time; CI, confidence interval; DI-GM, dietary index for the gut microbiota; GERD, gastroesophageal reflux disease; β, regression coefficient; BMI, body mass index; MVPA, moderate-to-vigorous physical activity.

**Supplementary Table 5 Bonferroni-significant plasma proteins associated with DI-GM score in multivariable linear regression models**

| **Protein name** | **Direction with higher DI-GM score** | **Beta (per 1-point higher DI-GM score)** | **95% CI** | ***P* value** | **FDR** | **Bonferroni-adjusted *P* value** |
| --- | --- | --- | --- | --- | --- | --- |
| LEP | Negative | -0.037 | -0.044, -0.031 | 4.19E-29 | 6.10E-26 | 1.22E-25 |
| OXT | Negative | -0.076 | -0.089, -0.063 | 5.66E-29 | 6.10E-26 | 1.65E-25 |
| ASGR1 | Negative | -0.015 | -0.018, -0.013 | 7.75E-29 | 6.10E-26 | 2.26E-25 |
| DDC | Positive | 0.026 | 0.021, 0.030 | 8.38E-29 | 6.10E-26 | 2.44E-25 |
| FABP4 | Negative | -0.025 | -0.030, -0.021 | 5.57E-27 | 3.24E-24 | 1.62E-23 |
| FGF21 | Negative | -0.060 | -0.071, -0.049 | 6.27E-26 | 3.04E-23 | 1.82E-22 |
| INHBC | Negative | -0.021 | -0.025, -0.017 | 1.25E-24 | 5.20E-22 | 3.64E-21 |
| CD99L2 | Negative | -0.012 | -0.014, -0.010 | 2.54E-24 | 9.23E-22 | 7.38E-21 |
| GUCA2A | Positive | 0.016 | 0.013, 0.019 | 2.85E-23 | 9.20E-21 | 8.28E-20 |
| STC1 | Negative | -0.021 | -0.025, -0.017 | 3.63E-23 | 1.06E-20 | 1.06E-19 |
| FSTL3 | Negative | -0.014 | -0.017, -0.011 | 9.48E-22 | 2.51E-19 | 2.76E-18 |
| CFB | Negative | -0.013 | -0.016, -0.010 | 1.77E-20 | 4.29E-18 | 5.14E-17 |
| IGSF9 | Negative | -0.032 | -0.038, -0.025 | 2.76E-20 | 6.18E-18 | 8.04E-17 |
| SLC39A5 | Negative | -0.023 | -0.029, -0.018 | 6.65E-20 | 1.38E-17 | 1.94E-16 |
| HGF | Negative | -0.015 | -0.018, -0.012 | 7.91E-20 | 1.53E-17 | 2.30E-16 |
| PLAT | Negative | -0.022 | -0.026, -0.017 | 2.74E-19 | 4.99E-17 | 7.98E-16 |
| NELL1 | Positive | 0.017 | 0.013, 0.021 | 2.52E-18 | 4.31E-16 | 7.33E-15 |
| CDHR2 | Negative | -0.030 | -0.037, -0.023 | 4.09E-18 | 6.62E-16 | 1.19E-14 |
| PI3 | Negative | -0.022 | -0.027, -0.017 | 1.64E-17 | 2.51E-15 | 4.76E-14 |
| BGLAP | Negative | -0.031 | -0.038, -0.024 | 3.38E-17 | 4.90E-15 | 9.84E-14 |
| IL1RN | Negative | -0.022 | -0.027, -0.017 | 3.54E-17 | 4.90E-15 | 1.03E-13 |
| APCS | Negative | -0.012 | -0.015, -0.009 | 3.90E-16 | 5.16E-14 | 1.13E-12 |
| DSG2 | Positive | 0.010 | 0.008, 0.013 | 4.92E-16 | 6.22E-14 | 1.43E-12 |
| CTSD | Negative | -0.014 | -0.018, -0.011 | 6.02E-16 | 7.30E-14 | 1.75E-12 |
| ACP5 | Negative | -0.013 | -0.016, -0.010 | 8.89E-16 | 1.03E-13 | 2.59E-12 |
| ADM | Negative | -0.010 | -0.013, -0.008 | 9.94E-16 | 1.11E-13 | 2.89E-12 |
| PON2 | Negative | -0.013 | -0.017, -0.010 | 1.46E-15 | 1.57E-13 | 4.24E-12 |
| PRSS8 | Negative | -0.015 | -0.019, -0.011 | 1.92E-15 | 2.00E-13 | 5.60E-12 |
| ERBB4 | Positive | 0.009 | 0.007, 0.012 | 2.39E-15 | 2.40E-13 | 6.95E-12 |
| CNTN1 | Positive | 0.010 | 0.008, 0.013 | 3.75E-15 | 3.64E-13 | 1.09E-11 |
| SERPINF1 | Negative | -0.008 | -0.011, -0.006 | 2.32E-14 | 2.18E-12 | 6.75E-11 |
| INHBB | Negative | -0.016 | -0.020, -0.012 | 2.90E-14 | 2.64E-12 | 8.43E-11 |
| RARRES2 | Negative | -0.018 | -0.022, -0.013 | 3.11E-14 | 2.74E-12 | 9.05E-11 |
| NTRK3 | Positive | 0.008 | 0.006, 0.010 | 4.82E-14 | 4.13E-12 | 1.40E-10 |
| IL18R1 | Negative | -0.012 | -0.015, -0.009 | 6.71E-14 | 5.58E-12 | 1.95E-10 |
| F9 | Negative | -0.007 | -0.008, -0.005 | 8.49E-14 | 6.86E-12 | 2.47E-10 |
| NPPC | Negative | -0.016 | -0.021, -0.012 | 1.67E-13 | 1.31E-11 | 4.85E-10 |
| CGREF1 | Negative | -0.015 | -0.019, -0.011 | 1.20E-12 | 9.16E-11 | 3.48E-09 |
| NPDC1 | Negative | -0.010 | -0.013, -0.008 | 1.24E-12 | 9.29E-11 | 3.62E-09 |
| IGFBP4 | Negative | -0.014 | -0.017, -0.010 | 1.39E-12 | 9.92E-11 | 4.05E-09 |
| CCL16 | Negative | -0.018 | -0.023, -0.013 | 1.40E-12 | 9.92E-11 | 4.07E-09 |
| PALM2 | Negative | -0.011 | -0.014, -0.008 | 1.77E-12 | 1.23E-10 | 5.16E-09 |
| HEPH | Positive | 0.008 | 0.006, 0.010 | 2.35E-12 | 1.54E-10 | 6.84E-09 |
| ORM1 | Negative | -0.006 | -0.007, -0.004 | 2.40E-12 | 1.54E-10 | 6.97E-09 |
| OSM | Negative | -0.024 | -0.031, -0.017 | 2.42E-12 | 1.54E-10 | 7.05E-09 |
| MXRA8 | Positive | 0.009 | 0.006, 0.011 | 2.46E-12 | 1.54E-10 | 7.15E-09 |
| MEGF10 | Positive | 0.014 | 0.010, 0.017 | 2.49E-12 | 1.54E-10 | 7.24E-09 |
| COL18A1 | Negative | -0.008 | -0.010, -0.006 | 3.05E-12 | 1.85E-10 | 8.88E-09 |
| CDH17 | Positive | 0.023 | 0.016, 0.029 | 4.12E-12 | 2.45E-10 | 1.20E-08 |
| SPON2 | Negative | -0.011 | -0.014, -0.008 | 4.77E-12 | 2.77E-10 | 1.39E-08 |
| EFNA1 | Negative | -0.009 | -0.011, -0.006 | 1.16E-11 | 6.62E-10 | 3.37E-08 |
| NT5E | Negative | -0.019 | -0.024, -0.013 | 1.67E-11 | 9.32E-10 | 4.85E-08 |
| CANT1 | Negative | -0.007 | -0.009, -0.005 | 2.53E-11 | 1.39E-09 | 7.36E-08 |
| SCLY | Negative | -0.015 | -0.020, -0.011 | 2.66E-11 | 1.43E-09 | 7.75E-08 |
| COQ7 | Negative | -0.012 | -0.016, -0.009 | 2.76E-11 | 1.46E-09 | 8.03E-08 |
| CLEC4D | Negative | -0.020 | -0.026, -0.014 | 3.04E-11 | 1.58E-09 | 8.84E-08 |
| TREM2 | Negative | -0.016 | -0.021, -0.011 | 3.38E-11 | 1.73E-09 | 9.85E-08 |
| GPR37 | Negative | -0.021 | -0.027, -0.014 | 4.24E-11 | 2.13E-09 | 1.24E-07 |
| LGALS1 | Negative | -0.011 | -0.014, -0.008 | 5.74E-11 | 2.83E-09 | 1.67E-07 |
| POLR2F | Negative | -0.011 | -0.014, -0.007 | 6.25E-11 | 3.03E-09 | 1.82E-07 |
| NOMO1 | Negative | -0.007 | -0.010, -0.005 | 8.20E-11 | 3.91E-09 | 2.39E-07 |
| CLMP | Negative | -0.006 | -0.008, -0.005 | 8.61E-11 | 4.04E-09 | 2.51E-07 |
| MZB1 | Negative | -0.016 | -0.021, -0.011 | 9.91E-11 | 4.58E-09 | 2.88E-07 |
| BST2 | Negative | -0.016 | -0.021, -0.011 | 1.27E-10 | 5.79E-09 | 3.70E-07 |
| MYOM3 | Positive | 0.025 | 0.017, 0.032 | 1.45E-10 | 6.47E-09 | 4.21E-07 |
| CHI3L1 | Negative | -0.023 | -0.030, -0.016 | 1.59E-10 | 7.01E-09 | 4.63E-07 |
| CNTN4 | Positive | 0.008 | 0.005, 0.010 | 1.69E-10 | 7.32E-09 | 4.91E-07 |
| FAM20A | Negative | -0.008 | -0.010, -0.005 | 1.72E-10 | 7.36E-09 | 5.00E-07 |
| ANG | Negative | -0.010 | -0.014, -0.007 | 1.77E-10 | 7.47E-09 | 5.15E-07 |
| CFI | Negative | -0.006 | -0.007, -0.004 | 2.02E-10 | 8.39E-09 | 5.87E-07 |
| LPL | Positive | 0.015 | 0.010, 0.019 | 2.25E-10 | 9.21E-09 | 6.54E-07 |
| AGRN | Negative | -0.010 | -0.013, -0.007 | 2.31E-10 | 9.33E-09 | 6.72E-07 |
| MMP8 | Negative | -0.023 | -0.030, -0.016 | 2.99E-10 | 1.19E-08 | 8.69E-07 |
| TGFA | Negative | -0.014 | -0.018, -0.009 | 3.03E-10 | 1.19E-08 | 8.81E-07 |
| VEGFB | Negative | -0.008 | -0.010, -0.005 | 3.20E-10 | 1.24E-08 | 9.30E-07 |
| GLA | Negative | -0.010 | -0.013, -0.007 | 3.23E-10 | 1.24E-08 | 9.39E-07 |
| RNASE4 | Negative | -0.007 | -0.009, -0.005 | 4.13E-10 | 1.56E-08 | 1.20E-06 |
| FAM3C | Negative | -0.009 | -0.012, -0.006 | 4.89E-10 | 1.82E-08 | 1.42E-06 |
| SELE | Negative | -0.015 | -0.020, -0.010 | 5.86E-10 | 2.16E-08 | 1.71E-06 |
| GDF15 | Negative | -0.012 | -0.015, -0.008 | 6.13E-10 | 2.23E-08 | 1.78E-06 |
| CD302 | Negative | -0.009 | -0.011, -0.006 | 8.40E-10 | 3.02E-08 | 2.44E-06 |
| EFNA4 | Negative | -0.008 | -0.011, -0.006 | 8.76E-10 | 3.11E-08 | 2.55E-06 |
| DDT | Negative | -0.011 | -0.014, -0.007 | 9.39E-10 | 3.29E-08 | 2.73E-06 |
| GHR | Negative | -0.007 | -0.010, -0.005 | 9.83E-10 | 3.41E-08 | 2.86E-06 |
| GGT1 | Negative | -0.014 | -0.019, -0.010 | 1.03E-09 | 3.52E-08 | 2.99E-06 |
| FGFR2 | Negative | -0.006 | -0.008, -0.004 | 1.05E-09 | 3.57E-08 | 3.07E-06 |
| RNASE6 | Negative | -0.008 | -0.010, -0.005 | 1.07E-09 | 3.57E-08 | 3.10E-06 |
| COCH | Negative | -0.012 | -0.016, -0.008 | 1.22E-09 | 4.02E-08 | 3.54E-06 |
| MPO | Negative | -0.014 | -0.018, -0.009 | 1.42E-09 | 4.64E-08 | 4.13E-06 |
| GFRA1 | Negative | -0.008 | -0.010, -0.005 | 1.59E-09 | 5.09E-08 | 4.62E-06 |
| CDHR5 | Negative | -0.011 | -0.015, -0.008 | 1.59E-09 | 5.09E-08 | 4.63E-06 |
| CCL3 | Negative | -0.017 | -0.022, -0.011 | 1.68E-09 | 5.26E-08 | 4.88E-06 |
| CNTN3 | Negative | -0.009 | -0.013, -0.006 | 1.68E-09 | 5.26E-08 | 4.89E-06 |
| CSTB | Negative | -0.013 | -0.017, -0.008 | 1.89E-09 | 5.86E-08 | 5.51E-06 |
| AOC3 | Positive | 0.009 | 0.006, 0.012 | 2.03E-09 | 6.21E-08 | 5.90E-06 |
| CST3 | Negative | -0.008 | -0.011, -0.005 | 2.27E-09 | 6.89E-08 | 6.62E-06 |
| PLAU | Positive | 0.008 | 0.005, 0.011 | 2.64E-09 | 7.88E-08 | 7.70E-06 |
| PTGDS | Negative | -0.008 | -0.011, -0.006 | 2.65E-09 | 7.88E-08 | 7.73E-06 |
| RELT | Negative | -0.008 | -0.010, -0.005 | 2.75E-09 | 8.08E-08 | 8.00E-06 |
| SELENOP | Positive | 0.006 | 0.004, 0.007 | 3.52E-09 | 1.02E-07 | 1.02E-05 |
| PRTG | Positive | 0.007 | 0.005, 0.009 | 3.53E-09 | 1.02E-07 | 1.03E-05 |
| HAO1 | Negative | -0.037 | -0.049, -0.025 | 3.57E-09 | 1.02E-07 | 1.04E-05 |
| BPIFB2 | Negative | -0.015 | -0.021, -0.010 | 3.60E-09 | 1.02E-07 | 1.05E-05 |
| RGMA | Positive | 0.009 | 0.006, 0.012 | 3.69E-09 | 1.03E-07 | 1.07E-05 |
| LILRA5 | Negative | -0.009 | -0.012, -0.006 | 3.87E-09 | 1.07E-07 | 1.13E-05 |
| HMOX2 | Negative | -0.013 | -0.017, -0.009 | 3.89E-09 | 1.07E-07 | 1.13E-05 |
| EPHA1 | Negative | -0.009 | -0.013, -0.006 | 4.05E-09 | 1.08E-07 | 1.18E-05 |
| COL4A1 | Positive | 0.011 | 0.007, 0.015 | 4.05E-09 | 1.08E-07 | 1.18E-05 |
| IL19 | Negative | -0.019 | -0.026, -0.013 | 4.05E-09 | 1.08E-07 | 1.18E-05 |
| IL10RB | Negative | -0.008 | -0.011, -0.005 | 4.09E-09 | 1.08E-07 | 1.19E-05 |
| TNFRSF1A | Negative | -0.008 | -0.010, -0.005 | 4.47E-09 | 1.17E-07 | 1.30E-05 |
| CALCA | Negative | -0.018 | -0.023, -0.012 | 4.51E-09 | 1.17E-07 | 1.31E-05 |
| ADAMTS15 | Negative | -0.013 | -0.017, -0.008 | 4.84E-09 | 1.25E-07 | 1.41E-05 |
| EGLN1 | Negative | -0.015 | -0.021, -0.010 | 7.06E-09 | 1.80E-07 | 2.05E-05 |
| RBP5 | Negative | -0.015 | -0.020, -0.010 | 8.01E-09 | 2.03E-07 | 2.33E-05 |
| LCAT | Negative | -0.006 | -0.007, -0.004 | 9.16E-09 | 2.30E-07 | 2.67E-05 |
| GUSB | Negative | -0.016 | -0.022, -0.011 | 9.75E-09 | 2.43E-07 | 2.84E-05 |
| NECTIN2 | Negative | -0.008 | -0.011, -0.005 | 1.09E-08 | 2.68E-07 | 3.17E-05 |
| GZMA | Negative | -0.010 | -0.013, -0.006 | 1.15E-08 | 2.82E-07 | 3.36E-05 |
| BOC | Positive | 0.007 | 0.004, 0.009 | 1.18E-08 | 2.86E-07 | 3.44E-05 |
| NCF2 | Negative | -0.024 | -0.032, -0.016 | 1.28E-08 | 3.08E-07 | 3.74E-05 |
| CCL23 | Negative | -0.011 | -0.015, -0.008 | 1.29E-08 | 3.08E-07 | 3.76E-05 |
| MAD1L1 | Negative | -0.013 | -0.017, -0.008 | 1.41E-08 | 3.33E-07 | 4.10E-05 |
| TMPRSS11D | Negative | -0.009 | -0.012, -0.006 | 1.63E-08 | 3.81E-07 | 4.73E-05 |
| CYTL1 | Negative | -0.006 | -0.008, -0.004 | 1.68E-08 | 3.91E-07 | 4.89E-05 |
| CD8A | Negative | -0.014 | -0.018, -0.009 | 1.80E-08 | 4.16E-07 | 5.24E-05 |
| COL6A3 | Negative | -0.009 | -0.012, -0.006 | 1.83E-08 | 4.20E-07 | 5.33E-05 |
| IGSF8 | Negative | -0.008 | -0.011, -0.005 | 1.86E-08 | 4.22E-07 | 5.40E-05 |
| NHLRC3 | Negative | -0.007 | -0.010, -0.005 | 2.26E-08 | 5.09E-07 | 6.57E-05 |
| MCAM | Positive | 0.009 | 0.006, 0.012 | 2.40E-08 | 5.37E-07 | 6.99E-05 |
| VSIG4 | Negative | -0.011 | -0.014, -0.007 | 2.53E-08 | 5.61E-07 | 7.35E-05 |
| CCL20 | Negative | -0.023 | -0.031, -0.015 | 2.61E-08 | 5.75E-07 | 7.59E-05 |
| PENK | Negative | -0.007 | -0.009, -0.004 | 2.63E-08 | 5.76E-07 | 7.66E-05 |
| SHISA5 | Negative | -0.006 | -0.008, -0.004 | 2.88E-08 | 6.25E-07 | 8.38E-05 |
| TIMP1 | Negative | -0.007 | -0.010, -0.005 | 3.10E-08 | 6.67E-07 | 9.01E-05 |
| LCN2 | Negative | -0.010 | -0.014, -0.006 | 3.14E-08 | 6.71E-07 | 9.13E-05 |
| PRCP | Negative | -0.008 | -0.011, -0.005 | 3.67E-08 | 7.79E-07 | 1.07E-04 |
| TGFBI | Negative | -0.007 | -0.010, -0.005 | 3.71E-08 | 7.82E-07 | 1.08E-04 |
| LGALS3BP | Negative | -0.007 | -0.010, -0.005 | 3.76E-08 | 7.86E-07 | 1.09E-04 |
| ITGAV | Positive | 0.004 | 0.003, 0.006 | 3.90E-08 | 8.11E-07 | 1.14E-04 |
| ADGRG2 | Positive | 0.007 | 0.005, 0.010 | 4.03E-08 | 8.32E-07 | 1.17E-04 |
| IGDCC4 | Positive | 0.006 | 0.004, 0.008 | 4.07E-08 | 8.35E-07 | 1.19E-04 |
| ASGR2 | Negative | -0.006 | -0.008, -0.004 | 4.28E-08 | 8.70E-07 | 1.24E-04 |
| VWC2 | Negative | -0.010 | -0.014, -0.007 | 4.32E-08 | 8.74E-07 | 1.26E-04 |
| IL6 | Negative | -0.019 | -0.026, -0.012 | 4.61E-08 | 9.26E-07 | 1.34E-04 |
| GOLM2 | Negative | -0.006 | -0.008, -0.004 | 4.67E-08 | 9.30E-07 | 1.36E-04 |
| HSPG2 | Negative | -0.007 | -0.009, -0.004 | 5.04E-08 | 9.98E-07 | 1.47E-04 |
| ACY1 | Negative | -0.016 | -0.021, -0.010 | 5.75E-08 | 1.13E-06 | 1.67E-04 |
| TAFA5 | Negative | -0.009 | -0.012, -0.006 | 7.82E-08 | 1.53E-06 | 2.28E-04 |
| CCL14 | Negative | -0.010 | -0.014, -0.007 | 8.15E-08 | 1.58E-06 | 2.37E-04 |
| ERN1 | Negative | -0.009 | -0.012, -0.006 | 8.78E-08 | 1.69E-06 | 2.56E-04 |
| OGN | Negative | -0.010 | -0.014, -0.007 | 8.94E-08 | 1.71E-06 | 2.60E-04 |
| CSF1 | Negative | -0.007 | -0.009, -0.004 | 9.79E-08 | 1.86E-06 | 2.85E-04 |
| GRPEL1 | Negative | -0.015 | -0.021, -0.010 | 1.14E-07 | 2.16E-06 | 3.33E-04 |
| MMP9 | Negative | -0.016 | -0.021, -0.010 | 1.17E-07 | 2.20E-06 | 3.40E-04 |
| CD300E | Negative | -0.010 | -0.014, -0.006 | 1.20E-07 | 2.24E-06 | 3.49E-04 |
| EPHB6 | Negative | -0.008 | -0.011, -0.005 | 1.32E-07 | 2.44E-06 | 3.83E-04 |
| ELOA | Negative | -0.014 | -0.019, -0.009 | 1.32E-07 | 2.44E-06 | 3.85E-04 |
| FURIN | Negative | -0.009 | -0.012, -0.005 | 1.33E-07 | 2.44E-06 | 3.88E-04 |
| TNFRSF10B | Negative | -0.009 | -0.013, -0.006 | 1.34E-07 | 2.44E-06 | 3.90E-04 |
| SCARB2 | Negative | -0.007 | -0.010, -0.005 | 1.39E-07 | 2.51E-06 | 4.04E-04 |
| CES1 | Negative | -0.020 | -0.027, -0.012 | 1.46E-07 | 2.62E-06 | 4.24E-04 |
| SERPIND1 | Negative | -0.006 | -0.009, -0.004 | 1.57E-07 | 2.80E-06 | 4.56E-04 |
| MME | Negative | -0.020 | -0.027, -0.012 | 1.76E-07 | 3.13E-06 | 5.13E-04 |
| F7 | Negative | -0.008 | -0.010, -0.005 | 1.77E-07 | 3.13E-06 | 5.16E-04 |
| LTBP3 | Negative | -0.014 | -0.019, -0.008 | 1.83E-07 | 3.21E-06 | 5.33E-04 |
| HYOU1 | Negative | -0.006 | -0.008, -0.003 | 1.89E-07 | 3.29E-06 | 5.50E-04 |
| BAIAP2 | Negative | -0.015 | -0.021, -0.010 | 1.93E-07 | 3.34E-06 | 5.61E-04 |
| RBP7 | Negative | -0.013 | -0.018, -0.008 | 1.97E-07 | 3.40E-06 | 5.75E-04 |
| LILRB4 | Negative | -0.011 | -0.015, -0.007 | 2.03E-07 | 3.48E-06 | 5.91E-04 |
| LGALS9 | Negative | -0.008 | -0.011, -0.005 | 2.06E-07 | 3.51E-06 | 6.01E-04 |
| MUC13 | Negative | -0.012 | -0.017, -0.008 | 2.14E-07 | 3.62E-06 | 6.23E-04 |
| COLEC12 | Negative | -0.006 | -0.009, -0.004 | 2.17E-07 | 3.65E-06 | 6.33E-04 |
| MNDA | Negative | -0.025 | -0.035, -0.016 | 2.18E-07 | 3.65E-06 | 6.35E-04 |
| REG4 | Negative | -0.012 | -0.016, -0.007 | 2.45E-07 | 4.08E-06 | 7.14E-04 |
| MET | Positive | 0.005 | 0.003, 0.007 | 2.61E-07 | 4.31E-06 | 7.59E-04 |
| TNFRSF10A | Negative | -0.008 | -0.011, -0.005 | 2.99E-07 | 4.92E-06 | 8.71E-04 |
| TNFRSF6B | Negative | -0.013 | -0.018, -0.008 | 3.31E-07 | 5.42E-06 | 9.64E-04 |
| PDZK1 | Negative | -0.009 | -0.013, -0.006 | 3.39E-07 | 5.51E-06 | 9.87E-04 |
| LAIR1 | Negative | -0.009 | -0.013, -0.006 | 3.43E-07 | 5.53E-06 | 9.98E-04 |
| CRHBP | Negative | -0.007 | -0.010, -0.004 | 3.46E-07 | 5.53E-06 | 1.01E-03 |
| KRT18 | Negative | -0.019 | -0.027, -0.012 | 3.46E-07 | 5.53E-06 | 1.01E-03 |
| LBR | Negative | -0.017 | -0.023, -0.010 | 3.69E-07 | 5.86E-06 | 1.07E-03 |
| SPINK6 | Negative | -0.014 | -0.019, -0.008 | 3.80E-07 | 6.01E-06 | 1.11E-03 |
| PRAP1 | Negative | -0.009 | -0.012, -0.005 | 3.86E-07 | 6.07E-06 | 1.12E-03 |
| ADAMTS8 | Positive | 0.011 | 0.007, 0.016 | 4.33E-07 | 6.78E-06 | 1.26E-03 |
| ECHDC3 | Negative | -0.015 | -0.021, -0.009 | 4.66E-07 | 7.25E-06 | 1.36E-03 |
| FUS | Negative | -0.010 | -0.013, -0.006 | 4.77E-07 | 7.38E-06 | 1.39E-03 |
| PXN | Negative | -0.013 | -0.018, -0.008 | 5.03E-07 | 7.74E-06 | 1.46E-03 |
| FGR | Negative | -0.014 | -0.019, -0.008 | 5.36E-07 | 8.20E-06 | 1.56E-03 |
| VNN1 | Negative | -0.015 | -0.021, -0.009 | 5.38E-07 | 8.20E-06 | 1.57E-03 |
| PGLYRP1 | Negative | -0.010 | -0.015, -0.006 | 5.54E-07 | 8.39E-06 | 1.61E-03 |
| CRELD1 | Negative | -0.008 | -0.011, -0.005 | 6.12E-07 | 9.23E-06 | 1.78E-03 |
| ENPP6 | Positive | 0.008 | 0.005, 0.010 | 6.21E-07 | 9.31E-06 | 1.81E-03 |
| SSC5D | Negative | -0.010 | -0.015, -0.006 | 6.57E-07 | 9.81E-06 | 1.91E-03 |
| UMOD | Positive | 0.013 | 0.008, 0.018 | 6.66E-07 | 9.88E-06 | 1.94E-03 |
| NOS3 | Negative | -0.012 | -0.017, -0.007 | 6.98E-07 | 1.03E-05 | 2.03E-03 |
| CD38 | Negative | -0.008 | -0.010, -0.005 | 7.20E-07 | 1.06E-05 | 2.09E-03 |
| ANXA2 | Negative | -0.014 | -0.020, -0.009 | 7.36E-07 | 1.08E-05 | 2.14E-03 |
| SORCS2 | Negative | -0.008 | -0.011, -0.005 | 7.59E-07 | 1.10E-05 | 2.21E-03 |
| VEGFA | Negative | -0.014 | -0.019, -0.008 | 8.12E-07 | 1.17E-05 | 2.36E-03 |
| CES2 | Negative | -0.013 | -0.019, -0.008 | 8.12E-07 | 1.17E-05 | 2.36E-03 |
| ITIH4 | Negative | -0.005 | -0.008, -0.003 | 8.50E-07 | 1.22E-05 | 2.47E-03 |
| CPM | Negative | -0.008 | -0.011, -0.005 | 8.59E-07 | 1.22E-05 | 2.50E-03 |
| RRM2 | Negative | -0.012 | -0.017, -0.007 | 8.62E-07 | 1.22E-05 | 2.51E-03 |
| CD59 | Negative | -0.005 | -0.007, -0.003 | 9.04E-07 | 1.27E-05 | 2.63E-03 |
| ACP6 | Negative | -0.017 | -0.023, -0.010 | 9.05E-07 | 1.27E-05 | 2.63E-03 |
| SMPDL3A | Negative | -0.016 | -0.022, -0.009 | 9.06E-07 | 1.27E-05 | 2.64E-03 |
| DKK4 | Negative | -0.011 | -0.015, -0.006 | 9.64E-07 | 1.34E-05 | 2.81E-03 |
| ACAA1 | Negative | -0.019 | -0.026, -0.011 | 9.80E-07 | 1.36E-05 | 2.85E-03 |
| SERPINB8 | Negative | -0.013 | -0.019, -0.008 | 1.01E-06 | 1.39E-05 | 2.94E-03 |
| C19ORF12 | Negative | -0.015 | -0.021, -0.009 | 1.05E-06 | 1.44E-05 | 3.05E-03 |
| APEX1 | Negative | -0.014 | -0.020, -0.008 | 1.17E-06 | 1.60E-05 | 3.41E-03 |
| ST6GAL1 | Negative | -0.007 | -0.010, -0.004 | 1.19E-06 | 1.62E-05 | 3.46E-03 |
| TNF | Negative | -0.009 | -0.012, -0.005 | 1.24E-06 | 1.67E-05 | 3.62E-03 |
| ITGA11 | Positive | 0.008 | 0.005, 0.011 | 1.24E-06 | 1.67E-05 | 3.62E-03 |
| CCL22 | Negative | -0.012 | -0.017, -0.007 | 1.34E-06 | 1.80E-05 | 3.91E-03 |
| PRTN3 | Negative | -0.011 | -0.016, -0.007 | 1.37E-06 | 1.83E-05 | 3.99E-03 |
| TNFRSF12A | Negative | -0.008 | -0.011, -0.005 | 1.49E-06 | 1.98E-05 | 4.33E-03 |
| FOLR1 | Positive | 0.006 | 0.004, 0.009 | 1.53E-06 | 2.02E-05 | 4.44E-03 |
| GAPDH | Negative | -0.006 | -0.009, -0.004 | 1.62E-06 | 2.13E-05 | 4.71E-03 |
| LIFR | Positive | 0.006 | 0.003, 0.008 | 1.66E-06 | 2.18E-05 | 4.84E-03 |
| HAVCR1 | Negative | -0.014 | -0.020, -0.008 | 1.67E-06 | 2.19E-05 | 4.87E-03 |
| IGFBP6 | Negative | -0.007 | -0.009, -0.004 | 1.68E-06 | 2.19E-05 | 4.90E-03 |
| RNASE1 | Negative | -0.005 | -0.007, -0.003 | 1.76E-06 | 2.27E-05 | 5.11E-03 |
| THBS2 | Negative | -0.008 | -0.012, -0.005 | 1.82E-06 | 2.34E-05 | 5.30E-03 |
| IGFBP2 | Positive | 0.014 | 0.008, 0.020 | 1.90E-06 | 2.43E-05 | 5.52E-03 |
| CXCL10 | Negative | -0.016 | -0.022, -0.009 | 1.95E-06 | 2.49E-05 | 5.68E-03 |
| MIA | Negative | -0.009 | -0.012, -0.005 | 2.00E-06 | 2.53E-05 | 5.82E-03 |
| S100A12 | Negative | -0.017 | -0.025, -0.010 | 2.00E-06 | 2.53E-05 | 5.83E-03 |
| APBB1IP | Negative | -0.011 | -0.015, -0.006 | 2.09E-06 | 2.63E-05 | 6.07E-03 |
| SOST | Negative | -0.009 | -0.013, -0.005 | 2.12E-06 | 2.66E-05 | 6.16E-03 |
| CLEC6A | Negative | -0.011 | -0.015, -0.006 | 2.18E-06 | 2.73E-05 | 6.35E-03 |
| CXCL13 | Negative | -0.014 | -0.020, -0.008 | 2.26E-06 | 2.82E-05 | 6.59E-03 |
| SERPINE1 | Negative | -0.016 | -0.022, -0.009 | 2.34E-06 | 2.90E-05 | 6.81E-03 |
| S100A11 | Negative | -0.011 | -0.015, -0.006 | 2.47E-06 | 3.05E-05 | 7.20E-03 |
| YAP1 | Negative | -0.005 | -0.007, -0.003 | 2.68E-06 | 3.29E-05 | 7.81E-03 |
| ACVRL1 | Negative | -0.005 | -0.008, -0.003 | 2.79E-06 | 3.42E-05 | 8.13E-03 |
| A1BG | Negative | -0.003 | -0.005, -0.002 | 2.83E-06 | 3.44E-05 | 8.23E-03 |
| AZU1 | Negative | -0.018 | -0.025, -0.010 | 3.03E-06 | 3.67E-05 | 8.81E-03 |
| XCL1 | Negative | -0.013 | -0.018, -0.007 | 3.07E-06 | 3.70E-05 | 8.92E-03 |
| HJV | Negative | -0.009 | -0.012, -0.005 | 3.12E-06 | 3.75E-05 | 9.07E-03 |
| IL7R | Positive | 0.012 | 0.007, 0.017 | 3.14E-06 | 3.76E-05 | 9.13E-03 |
| IFNLR1 | Negative | -0.009 | -0.012, -0.005 | 3.32E-06 | 3.96E-05 | 9.67E-03 |
| TPP1 | Negative | -0.010 | -0.014, -0.006 | 3.35E-06 | 3.98E-05 | 9.76E-03 |
| LTBR | Negative | -0.006 | -0.009, -0.004 | 3.37E-06 | 3.99E-05 | 9.81E-03 |
| MIF | Negative | -0.016 | -0.022, -0.009 | 3.46E-06 | 4.07E-05 | 1.01E-02 |
| DPY30 | Negative | -0.013 | -0.019, -0.008 | 3.72E-06 | 4.36E-05 | 1.08E-02 |
| TIGAR | Negative | -0.011 | -0.015, -0.006 | 3.79E-06 | 4.43E-05 | 1.10E-02 |
| CD27 | Negative | -0.009 | -0.013, -0.005 | 3.80E-06 | 4.43E-05 | 1.11E-02 |
| GRN | Negative | -0.005 | -0.008, -0.003 | 3.83E-06 | 4.44E-05 | 1.11E-02 |
| RETN | Negative | -0.009 | -0.013, -0.005 | 3.87E-06 | 4.45E-05 | 1.12E-02 |
| SMAD5 | Negative | -0.005 | -0.007, -0.003 | 3.87E-06 | 4.45E-05 | 1.13E-02 |
| CRELD2 | Negative | -0.012 | -0.017, -0.007 | 3.93E-06 | 4.51E-05 | 1.14E-02 |
| TNFRSF17 | Negative | -0.008 | -0.011, -0.004 | 4.25E-06 | 4.85E-05 | 1.24E-02 |
| TNFSF14 | Negative | -0.013 | -0.019, -0.008 | 4.32E-06 | 4.91E-05 | 1.26E-02 |
| THOP1 | Negative | -0.007 | -0.010, -0.004 | 4.37E-06 | 4.95E-05 | 1.27E-02 |
| NCAM2 | Positive | 0.006 | 0.004, 0.009 | 4.59E-06 | 5.17E-05 | 1.33E-02 |
| CFC1 | Negative | -0.011 | -0.015, -0.006 | 4.69E-06 | 5.27E-05 | 1.36E-02 |
| SH2D1A | Negative | -0.013 | -0.018, -0.007 | 4.85E-06 | 5.43E-05 | 1.41E-02 |
| GPKOW | Negative | -0.006 | -0.009, -0.004 | 4.88E-06 | 5.44E-05 | 1.42E-02 |
| TGFBR2 | Negative | -0.007 | -0.010, -0.004 | 4.98E-06 | 5.53E-05 | 1.45E-02 |
| PTS | Negative | -0.012 | -0.017, -0.007 | 5.04E-06 | 5.57E-05 | 1.47E-02 |
| CA5A | Negative | -0.020 | -0.028, -0.011 | 5.23E-06 | 5.76E-05 | 1.52E-02 |
| RASSF2 | Negative | -0.012 | -0.017, -0.007 | 5.36E-06 | 5.89E-05 | 1.56E-02 |
| SMNDC1 | Negative | -0.012 | -0.017, -0.007 | 5.42E-06 | 5.93E-05 | 1.58E-02 |
| C1R | Negative | -0.004 | -0.005, -0.002 | 5.54E-06 | 6.03E-05 | 1.61E-02 |
| IL1RAP | Positive | 0.010 | 0.006, 0.014 | 5.56E-06 | 6.03E-05 | 1.62E-02 |
| RSPO1 | Negative | -0.008 | -0.011, -0.004 | 5.65E-06 | 6.11E-05 | 1.64E-02 |
| SEMA3F | Negative | -0.006 | -0.008, -0.003 | 5.83E-06 | 6.29E-05 | 1.70E-02 |
| CHCHD10 | Negative | -0.008 | -0.011, -0.004 | 5.90E-06 | 6.33E-05 | 1.72E-02 |
| WFDC2 | Negative | -0.008 | -0.011, -0.004 | 5.93E-06 | 6.33E-05 | 1.72E-02 |
| EIF4EBP1 | Negative | -0.015 | -0.021, -0.008 | 5.94E-06 | 6.33E-05 | 1.73E-02 |
| FOXO3 | Negative | -0.021 | -0.030, -0.012 | 6.11E-06 | 6.48E-05 | 1.78E-02 |
| AGXT | Negative | -0.017 | -0.024, -0.009 | 6.14E-06 | 6.48E-05 | 1.79E-02 |
| SRP14 | Negative | -0.013 | -0.019, -0.008 | 6.15E-06 | 6.48E-05 | 1.79E-02 |
| MELTF | Positive | 0.006 | 0.003, 0.008 | 6.17E-06 | 6.48E-05 | 1.79E-02 |
| CEACAM16 | Positive | 0.016 | 0.009, 0.022 | 6.46E-06 | 6.76E-05 | 1.88E-02 |
| CD74 | Negative | -0.008 | -0.011, -0.004 | 6.92E-06 | 7.21E-05 | 2.01E-02 |
| C1S | Negative | -0.004 | -0.006, -0.002 | 7.17E-06 | 7.46E-05 | 2.09E-02 |
| CXCL17 | Negative | -0.009 | -0.013, -0.005 | 7.65E-06 | 7.92E-05 | 2.23E-02 |
| CKAP4 | Negative | -0.006 | -0.008, -0.003 | 7.68E-06 | 7.93E-05 | 2.24E-02 |
| NMNAT1 | Negative | -0.016 | -0.022, -0.009 | 7.85E-06 | 8.08E-05 | 2.29E-02 |
| FCN1 | Negative | -0.011 | -0.016, -0.006 | 8.02E-06 | 8.21E-05 | 2.33E-02 |
| CREG1 | Negative | -0.008 | -0.012, -0.005 | 8.68E-06 | 8.83E-05 | 2.53E-02 |
| PROC | Negative | -0.006 | -0.008, -0.003 | 8.69E-06 | 8.83E-05 | 2.53E-02 |
| ALDH1A1 | Negative | -0.012 | -0.017, -0.006 | 8.71E-06 | 8.83E-05 | 2.53E-02 |
| CKB | Positive | 0.011 | 0.006, 0.016 | 9.47E-06 | 9.57E-05 | 2.76E-02 |
| CALB2 | Negative | -0.006 | -0.009, -0.004 | 9.51E-06 | 9.57E-05 | 2.77E-02 |
| LY6D | Negative | -0.008 | -0.011, -0.004 | 9.55E-06 | 9.58E-05 | 2.78E-02 |
| RIDA | Negative | -0.009 | -0.013, -0.005 | 9.91E-06 | 9.91E-05 | 2.88E-02 |
| AREG | Negative | -0.010 | -0.014, -0.005 | 1.01E-05 | 1.01E-04 | 2.94E-02 |
| BID | Negative | -0.015 | -0.021, -0.008 | 1.03E-05 | 1.02E-04 | 3.00E-02 |
| GAS6 | Positive | 0.005 | 0.003, 0.008 | 1.09E-05 | 1.07E-04 | 3.16E-02 |
| NUCB2 | Negative | -0.008 | -0.012, -0.005 | 1.12E-05 | 1.11E-04 | 3.26E-02 |
| OCLN | Negative | -0.008 | -0.011, -0.004 | 1.16E-05 | 1.14E-04 | 3.37E-02 |
| MCEMP1 | Negative | -0.016 | -0.024, -0.009 | 1.16E-05 | 1.14E-04 | 3.38E-02 |
| RTN4R | Negative | -0.007 | -0.010, -0.004 | 1.18E-05 | 1.15E-04 | 3.44E-02 |
| AMBP | Negative | -0.004 | -0.006, -0.002 | 1.18E-05 | 1.15E-04 | 3.44E-02 |
| PTP4A3 | Negative | -0.009 | -0.014, -0.005 | 1.28E-05 | 1.24E-04 | 3.73E-02 |
| TNFRSF1B | Negative | -0.007 | -0.011, -0.004 | 1.34E-05 | 1.30E-04 | 3.90E-02 |
| PLTP | Positive | 0.007 | 0.004, 0.011 | 1.39E-05 | 1.34E-04 | 4.06E-02 |
| ESM1 | Positive | 0.007 | 0.004, 0.011 | 1.44E-05 | 1.38E-04 | 4.19E-02 |
| DNPH1 | Negative | -0.011 | -0.016, -0.006 | 1.45E-05 | 1.39E-04 | 4.22E-02 |
| NTF3 | Positive | 0.007 | 0.004, 0.011 | 1.48E-05 | 1.41E-04 | 4.30E-02 |
| SELP | Negative | -0.012 | -0.018, -0.007 | 1.52E-05 | 1.45E-04 | 4.42E-02 |
| CASP1 | Negative | -0.014 | -0.021, -0.008 | 1.53E-05 | 1.45E-04 | 4.47E-02 |
| DNMBP | Negative | -0.013 | -0.019, -0.007 | 1.61E-05 | 1.52E-04 | 4.67E-02 |
| FABP3 | Negative | -0.009 | -0.013, -0.005 | 1.62E-05 | 1.52E-04 | 4.71E-02 |
| ZBTB17 | Negative | -0.009 | -0.012, -0.005 | 1.63E-05 | 1.53E-04 | 4.75E-02 |
| PLA2G10 | Positive | 0.012 | 0.007, 0.018 | 1.66E-05 | 1.55E-04 | 4.83E-02 |
| PGF | Negative | -0.005 | -0.007, -0.003 | 1.69E-05 | 1.58E-04 | 4.93E-02 |

Abbreviations: DI-GM, dietary index for gut microbiota.

**Supplementary Table 6** **FDR-significant plasma proteins associated with incident GERD in fully adjusted Cox regression models**

| **Protein name** | **GERD risk direction** | **HR (95% CI)** | ***P* value** | **FDR** | **Bonferroni-adjusted *P* value** |
| --- | --- | --- | --- | --- | --- |
| CHGA | Higher risk | 1.249 (1.172, 1.331) | 8.77E-12 | 2.55E-08 | 2.55E-08 |
| GAST | Higher risk | 1.128 (1.081, 1.178) | 3.39E-08 | 4.93E-05 | 9.86E-05 |
| MYOC | Lower risk | 0.777 (0.702, 0.859) | 9.55E-07 | 8.67E-04 | 2.78E-03 |
| PGA4 | Higher risk | 1.247 (1.141, 1.363) | 1.19E-06 | 8.67E-04 | 3.47E-03 |
| CNTN1 | Lower risk | 0.674 (0.572, 0.795) | 2.91E-06 | 1.69E-03 | 8.47E-03 |
| NTRK3 | Lower risk | 0.619 (0.501, 0.765) | 9.28E-06 | 4.50E-03 | 2.70E-02 |
| CNTN4 | Lower risk | 0.653 (0.539, 0.792) | 1.52E-05 | 5.42E-03 | 4.41E-02 |
| PLTP | Lower risk | 0.753 (0.662, 0.856) | 1.53E-05 | 5.42E-03 | 4.46E-02 |
| THBS2 | Lower risk | 0.748 (0.655, 0.854) | 1.68E-05 | 5.42E-03 | 4.87E-02 |
| ADAMTS13 | Lower risk | 0.658 (0.542, 0.799) | 2.37E-05 | 6.90E-03 | 6.90E-02 |
| NCAM2 | Lower risk | 0.694 (0.585, 0.824) | 2.83E-05 | 7.44E-03 | 8.23E-02 |
| NOTCH3 | Lower risk | 0.744 (0.647, 0.855) | 3.07E-05 | 7.44E-03 | 8.92E-02 |
| VSIG2 | Higher risk | 1.226 (1.113, 1.350) | 3.70E-05 | 8.29E-03 | 1.08E-01 |
| TNFRSF4 | Higher risk | 1.289 (1.140, 1.457) | 5.03E-05 | 1.03E-02 | 1.47E-01 |
| ADAMTS8 | Lower risk | 0.812 (0.733, 0.898) | 5.32E-05 | 1.03E-02 | 1.55E-01 |
| CLSTN2 | Lower risk | 0.787 (0.695, 0.891) | 1.54E-04 | 2.80E-02 | 4.48E-01 |
| LBP | Higher risk | 1.163 (1.075, 1.259) | 1.75E-04 | 2.99E-02 | 5.09E-01 |
| LEP | Higher risk | 1.137 (1.061, 1.220) | 2.98E-04 | 4.81E-02 | 8.66E-01 |
| CHRDL2 | Lower risk | 0.852 (0.780, 0.929) | 3.23E-04 | 4.95E-02 | 9.40E-01 |

Abbreviations: DI-GM, dietary index for gut microbiota.

**Supplementary Table 7 Shared proteins that were significant in both DI-GM score-associated proteomic analysis and GERD Cox analysis at FDR < 0.05**

| **Protein name** | **Direction with higher DI-GM score** | **Beta (95% CI) for DI-GM score** | **DI-GM *P* value** | **DI-GM FDR** | **GERD risk direction** | **HR (95% CI) for GERD** | **GERD *P* value** | **GERD FDR** |
| --- | --- | --- | --- | --- | --- | --- | --- | --- |
| MYOC | Positive | 0.006 (0.002, 0.011) | 3.97E-03 | 1.56E-02 | Lower risk | 0.777 (0.702, 0.859) | 9.55E-07 | 8.67E-04 |
| CNTN1 | Positive | 0.010 (0.008, 0.013) | 3.75E-15 | 3.64E-13 | Lower risk | 0.674 (0.572, 0.795) | 2.91E-06 | 1.69E-03 |
| NTRK3 | Positive | 0.008 (0.006, 0.010) | 4.82E-14 | 4.13E-12 | Lower risk | 0.619 (0.501, 0.765) | 9.28E-06 | 4.50E-03 |
| CNTN4 | Positive | 0.008 (0.005, 0.010) | 1.69E-10 | 7.32E-09 | Lower risk | 0.653 (0.539, 0.792) | 1.52E-05 | 5.42E-03 |
| PLTP | Positive | 0.007 (0.004, 0.011) | 1.39E-05 | 1.34E-04 | Lower risk | 0.753 (0.662, 0.856) | 1.53E-05 | 5.42E-03 |
| THBS2 | Negative | -0.008 (-0.012, -0.005) | 1.82E-06 | 2.34E-05 | Lower risk | 0.748 (0.655, 0.854) | 1.68E-05 | 5.42E-03 |
| ADAMTS13 | Positive | 0.004 (0.001, 0.006) | 1.27E-03 | 6.29E-03 | Lower risk | 0.658 (0.542, 0.799) | 2.37E-05 | 6.90E-03 |
| NCAM2 | Positive | 0.006 (0.004, 0.009) | 4.59E-06 | 5.17E-05 | Lower risk | 0.694 (0.585, 0.824) | 2.83E-05 | 7.44E-03 |
| NOTCH3 | Positive | 0.007 (0.004, 0.010) | 1.81E-05 | 1.66E-04 | Lower risk | 0.744 (0.647, 0.855) | 3.07E-05 | 7.44E-03 |
| TNFRSF4 | Negative | -0.006 (-0.010, -0.003) | 4.24E-04 | 2.52E-03 | Higher risk | 1.289 (1.140, 1.457) | 5.03E-05 | 1.03E-02 |
| ADAMTS8 | Positive | 0.011 (0.007, 0.016) | 4.33E-07 | 6.78E-06 | Lower risk | 0.812 (0.733, 0.898) | 5.32E-05 | 1.03E-02 |
| LBP | Negative | -0.011 (-0.017, -0.005) | 1.13E-04 | 8.21E-04 | Higher risk | 1.163 (1.075, 1.259) | 1.75E-04 | 2.99E-02 |
| LEP | Negative | -0.037 (-0.044, -0.031) | 4.19E-29 | 6.10E-26 | Higher risk | 1.137 (1.061, 1.220) | 2.98E-04 | 4.81E-02 |

Abbreviations: DI-GM, dietary index for gut microbiota.

**Supplementary Table 8 GO enrichment results for the nine shared proteins positively associated with DI-GM score**

| **Protein set** | **ID** | **Description** | **GeneRatio** | **BgRatio** | ***P* value** | **Adjusted *P* value** | **q value** | **Count** |
| --- | --- | --- | --- | --- | --- | --- | --- | --- |
| Shared proteins positively associated with DI-GM score | GO:0048699 | generation of neurons | 6/9 | 349/2760 | 3.97E-02 | 3.97E-02 | 3.97E-02 | 6 |
| Shared proteins positively associated with DI-GM score | GO:0007158 | neuron cell-cell adhesion | 2/9 | 7/2760 | 3.97E-02 | 3.97E-02 | 3.97E-02 | 2 |
| Shared proteins positively associated with DI-GM score | GO:0030182 | neuron differentiation | 6/9 | 335/2760 | 3.97E-02 | 3.97E-02 | 3.97E-02 | 6 |
| Shared proteins positively associated with DI-GM score | GO:0007413 | axonal fasciculation | 2/9 | 10/2760 | 4.24E-02 | 4.24E-02 | 4.24E-02 | 2 |
| Shared proteins positively associated with DI-GM score | GO:0106030 | neuron projection fasciculation | 2/9 | 10/2760 | 4.24E-02 | 4.24E-02 | 4.24E-02 | 2 |
| Shared proteins positively associated with DI-GM score | GO:0022008 | neurogenesis | 6/9 | 414/2760 | 4.99E-02 | 4.99E-02 | 4.99E-02 | 6 |
| Shared proteins positively associated with DI-GM score | GO:0031175 | neuron projection development | 5/9 | 263/2760 | 4.99E-02 | 4.99E-02 | 4.99E-02 | 5 |

Abbreviations: DI-GM, dietary index for gut microbiota.

**Supplementary Table 9 Association between DI-GM and risk of incident GERD among participants who completed at least two 24-hour dietary recalls**

| **Characteristics** | **Case/total (%)** | **GERD** | | | | |
| --- | --- | --- | --- | --- | --- | --- |
|  |  | **Model 1** | |  | **Model 2** | |
|  |  | **HR (95% CI)** | ***P* value** |  | **HR (95% CI)** | ***P* value** |
| DI-GM | 7204/82340 (8.7) | 0.952 (0.941-0.963) | <0.001 |  | 0.966 (0.954-0.977) | <0.001 |
| DI-GM group |  |  |  |  |  |  |
| 0-3 | 1558/16319 (9.5) | Ref |  |  | Ref |  |
| 4 | 1319/14168 (9.3) | 0.941 (0.874-1.012) | 0.103 |  | 0.969 (0.900-1.043) | 0.401 |
| 5 | 1382/15806 (8.7) | 0.869 (0.808-0.935) | <0.001 |  | 0.909 (0.845-0.978) | 0.010 |
| ≥6 | 2945/36047 (8.2) | 0.790 (0.742-0.840) | <0.001 |  | 0.847 (0.795-0.902) | <0.001 |
| *P* for trend |  |  | <0.001 |  |  | <0.001 |
| Beneficial to gut microbiota | 7204/82340 (8.7) | 0.943 (0.930-0.956) | <0.001 |  | 0.959 (0.946-0.972) | <0.001 |
| Unfavorable to gut microbiota | 7204/82340 (8.7) | 0.964 (0.938-0.990) | 0.007 |  | 0.976 (0.950-1.003) | 0.076 |

Note: Model 1 was adjusted for age and sex. Model 2 was based on Model 1 and further adjusted for ethnicity, Townsend deprivation index, education, smoking status, alcohol status, MVPA, CVD, diabetes, hypertension, anxiety, depression, and sleep disorder.

Abbreviations: DI-GM, dietary index for gut microbiota; GERD, gastroesophageal reflux disease; MVPA, moderate-to-vigorous physical activity; CVD, cardiovascular disease.

**Supplementary Table 10 Association between DI-GM and risk of incident GERD after excluding GERD cases within the first two years of follow-up**

| **Characteristics** | **Case/total (%)** | **GERD** | | | | |
| --- | --- | --- | --- | --- | --- | --- |
|  |  | **Model 1** | |  | **Model 2** | |
|  |  | **HR (95% CI)** | ***P* value** |  | **HR (95% CI)** | ***P* value** |
| DI-GM | 11144/132788 (8.4) | 0.944 (0.935-0.953) | <0.001 |  | 0.958 (0.949-0.968) | <0.001 |
| DI-GM group |  |  |  |  |  |  |
| 0-3 | 2582/28195 (9.2) | Ref |  |  | Ref |  |
| 4 | 2224/24814 (9.0) | 0.945 (0.893-1.001) | 0.053 |  | 0.971 (0.917-1.028) | 0.308 |
| 5 | 2263/26683 (8.5) | 0.878 (0.830-0.929) | <0.001 |  | 0.915 (0.865-0.969) | 0.002 |
| ≥6 | 4075/53096 (7.7) | 0.768 (0.731-0.807) | <0.001 |  | 0.822 (0.782-0.865) | <0.001 |
| *P* for trend |  |  | <0.001 |  |  | <0.001 |
| Beneficial to gut microbiota | 11144/132788 (8.4) | 0.931 (0.921-0.942) | <0.001 |  | 0.948 (0.937-0.959) | <0.001 |
| Unfavorable to gut microbiota | 11144/132788 (8.4) | 0.978 (0.958-0.999) | 0.043 |  | 0.985 (0.965-1.007) | 0.174 |

Note: Model 1 was adjusted for age and sex. Model 2 was based on Model 1 and further adjusted for ethnicity, Townsend deprivation index, education, smoking status, alcohol status, MVPA, CVD, diabetes, hypertension, anxiety, depression, and sleep disorder.

Abbreviations: DI-GM, dietary index for gut microbiota; GERD, gastroesophageal reflux disease; MVPA, moderate-to-vigorous physical activity; CVD, cardiovascular disease.

**Supplementary Table 11 Association between DI-GM and risk of incident GERD using the date of the dietary assessment as baseline**

| **Characteristics** | **Case/total (%)** | **GERD** | | | | |
| --- | --- | --- | --- | --- | --- | --- |
|  |  | **Model 1** | |  | **Model 2** | |
|  |  | **HR (95% CI)** | ***P* value** |  | **HR (95% CI)** | ***P* value** |
| DI-GM | 11228/131641 (8.5) | 0.942 (0.933-0.951) | <0.001 |  | 0.956 (0.947-0.966) | <0.001 |
| DI-GM group |  |  |  |  |  |  |
| 0-3 | 2622/27955 (9.4) | Ref |  |  | Ref |  |
| 4 | 2241/24597 (9.1) | 0.937 (0.885-0.991) | 0.023 |  | 0.961 (0.909-1.017) | 0.172 |
| 5 | 2260/26456 (8.5) | 0.864 (0.816-0.914) | <0.001 |  | 0.900 (0.851-0.952) | <0.001 |
| ≥6 | 4105/52633 (7.8) | 0.761 (0.724-0.800) | <0.001 |  | 0.815 (0.776-0.857) | <0.001 |
| *P* for trend |  |  | <0.001 |  |  | <0.001 |
| Beneficial to gut microbiota | 11228/131641 (8.5) | 0.930 (0.919-0.941) | <0.001 |  | 0.947 (0.936-0.958) | <0.001 |
| Unfavorable to gut microbiota | 11228/131641 (8.5) | 0.974 (0.954-0.995) | 0.016 |  | 0.981 (0.961-1.002) | 0.083 |

Note: Model 1 was adjusted for age and sex. Model 2 was based on Model 1 and further adjusted for ethnicity, Townsend deprivation index, education, smoking status, alcohol status, MVPA, CVD, diabetes, hypertension, anxiety, depression, and sleep disorder.

Abbreviations: DI-GM, dietary index for gut microbiota; GERD, gastroesophageal reflux disease; MVPA, moderate-to-vigorous physical activity; CVD, cardiovascular disease.

**Supplementary Table 12 Association between DI-GM and risk of incident GERD, additionally adjusted for total energy intake**

| **Characteristics** | **Case/total (%)** | **GERD** | | | | |
| --- | --- | --- | --- | --- | --- | --- |
|  |  | **Model 1** | |  | **Model 2** | |
|  |  | **HR (95% CI)** | ***P* value** |  | **HR (95% CI)** | ***P* value** |
| DI-GM | 12271/133915 (9.2) | 0.942 (0.933-0.951) | <0.001 |  | 0.956 (0.947-0.965) | <0.001 |
| DI-GM group |  |  |  |  |  |  |
| 0-3 | 2882/28495 (10.1) | Ref |  |  | Ref |  |
| 4 | 2434/25024 (9.7) | 0.928 (0.879-0.979) | 0.006 |  | 0.953 (0.903-1.006) | 0.080 |
| 5 | 2479/26899 (9.2) | 0.863 (0.817-0.910) | <0.001 |  | 0.899 (0.852-0.949) | <0.001 |
| ≥6 | 4476/53497 (8.4) | 0.758 (0.723-0.794) | <0.001 |  | 0.811 (0.773-0.851) | <0.001 |
| *P* for trend |  |  | <0.001 |  |  | <0.001 |
| Beneficial to gut microbiota | 12271/133915 (9.2) | 0.930 (0.920-0.940) | <0.001 |  | 0.943 (0.932-0.954) | <0.001 |
| Unfavorable to gut microbiota | 12271/133915 (9.2) | 0.972 (0.952-0.992) | 0.006 |  | 0.977 (0.956-0.998) | 0.035 |

Note: Model 1 was adjusted for age and sex. Model 2 was based on Model 1 and further adjusted for ethnicity, Townsend deprivation index, education, smoking status, alcohol status, MVPA, total energy intake, CVD, diabetes, hypertension, anxiety, depression, and sleep disorder.

Abbreviations: DI-GM, dietary index for gut microbiota; GERD, gastroesophageal reflux disease; MVPA, moderate-to-vigorous physical activity; CVD, cardiovascular disease.

**Supplementary Table 13 Association between DI-GM and risk of incident GERD in a** **stratified Cox model**

| **Characteristics** | **Case/total (%)** | **GERD** | | | | |
| --- | --- | --- | --- | --- | --- | --- |
|  |  | **Model 1** | |  | **Model 2** | |
|  |  | **HR (95% CI)** | ***P* value** |  | **HR (95% CI)** | ***P* value** |
| DI-GM | 12271/133915 (9.2) | 0.942 (0.933-0.951) | <0.001 |  | 0.956 (0.947-0.965) | <0.001 |
| DI-GM group |  |  |  |  |  |  |
| 0-3 | 2882/28495 (10.1) | Ref |  |  | Ref |  |
| 4 | 2434/25024 (9.7) | 0.928 (0.879-0.979) | 0.006 |  | 0.953 (0.903-1.006) | 0.080 |
| 5 | 2479/26899 (9.2) | 0.863 (0.817-0.910) | <0.001 |  | 0.899 (0.852-0.949) | <0.001 |
| ≥6 | 4476/53497 (8.4) | 0.758 (0.723-0.794) | <0.001 |  | 0.811 (0.774-0.851) | <0.001 |
| *P* for trend |  |  | <0.001 |  |  | <0.001 |
| Beneficial to gut microbiota | 12271/133915 (9.2) | 0.930 (0.920-0.940) | <0.001 |  | 0.947 (0.936-0.957) | <0.001 |
| Unfavorable to gut microbiota | 12271/133915 (9.2) | 0.972 (0.952-0.992) | 0.006 |  | 0.979 (0.959-0.999) | 0.038 |

Note: Model 1 was adjusted for age and sex. Model 2 was based on Model 1 and further adjusted for ethnicity, Townsend deprivation index, education, smoking status, alcohol status, MVPA, CVD, diabetes, hypertension, and anxiety, with depression and sleep disorder included as stratification variables.

Abbreviations: DI-GM, dietary index for gut microbiota; GERD, gastroesophageal reflux disease; MVPA, moderate-to-vigorous physical activity; CVD, cardiovascular disease.

**Supplementary Table 14 Association between DI-GM and risk of incident GERD accounting for the competing risk of death**

| **Characteristics** | **GERD** | | | | |
| --- | --- | --- | --- | --- | --- |
|  | **Model 1** | |  | **Model 2** | |
|  | **sHR (95% CI)** | ***P* value** |  | **sHR (95% CI)** | ***P* value** |
| DI-GM | 0.944 (0.935-0.952) | <0.001 |  | 0.957 (0.948-0.966) | <0.001 |
| DI-GM group |  |  |  |  |  |
| 0-3 | Ref |  |  | Ref |  |
| 4 | 0.931 (0.882-0.983) | 0.010 |  | 0.956 (0.905-1.009) | 0.102 |
| 5 | 0.866 (0.820-0.914) | <0.001 |  | 0.901 (0.854-0.951) | <0.001 |
| ≥6 | 0.764 (0.729-0.801) | <0.001 |  | 0.817 (0.779-0.857) | <0.001 |
| *P* for trend |  | <0.001 |  |  | <0.001 |
| Beneficial to gut microbiota | 0.933 (0.922-0.943) | <0.001 |  | 0.949 (0.938-0.959) | <0.001 |
| Unfavorable to gut microbiota | 0.971 (0.952-0.991) | 0.005 |  | 0.978 (0.959-0.998) | 0.035 |

Note: Effect estimates are subdistribution hazard ratios (sHRs) from Fine-Gray models. Model 1 was adjusted for age and sex. Model 2 was based on Model 1 and further adjusted for ethnicity, Townsend deprivation index, education, smoking status, alcohol status, MVPA, CVD, diabetes, hypertension, anxiety, depression, and sleep disorder.

Abbreviations: DI-GM, dietary index for gut microbiota; GERD, gastroesophageal reflux disease; MVPA, moderate-to-vigorous physical activity; CVD, cardiovascular disease.

**Supplementary Table 15 Comparison of mean-imputed and protein-specific complete-case proteomic analyses**

| **Analysis measure** | **Mean-imputed analysis** | **Complete-case analysis** | **Agreement** |
| --- | --- | --- | --- |
| DI-GM-protein associations: FDR-significant proteins | 1,057 | 1,037 | 1,032 proteins overlapped |
| DI-GM-protein associations: Bonferroni-significant proteins | 312 | 310 | - |
| Correlation of DI-GM effect estimates | - | - | r = 0.997 |
| Protein-GERD associations: FDR-significant proteins | 19 | 19 | 18 proteins overlapped |
| Protein-GERD associations: Bonferroni-significant proteins | 9 | 10 | - |
| Correlation of protein-GERD Cox coefficients | - | - | r = 0.999 |
| Shared FDR-significant proteins | 13 | 14 | All 13 primary shared proteins retained |

FDR was controlled using the Benjamini-Hochberg method. Complete-case analyses used the non-missing observations available for each individual protein and did not impute protein values. The complete-case analysis identified one additional shared protein, APCS.

**Supplementary Table 16 Shared proteins identified in the protein-specific complete-case sensitivity analysis**

| **Protein** | **DI-GM beta (95% CI)** | **DI-GM FDR** | **GERD HR (95% CI)** | **GERD FDR** | **Directionally concordant** |
| --- | --- | --- | --- | --- | --- |
| MYOC | 0.007 (0.002 to 0.011) | 1.43E-02 | 0.776 (0.701 to 0.859) | 7.21E-04 | Yes |
| CNTN1 | 0.011 (0.008 to 0.013) | 3.08E-13 | 0.670 (0.568 to 0.790) | 1.16E-03 | Yes |
| NTRK3 | 0.008 (0.006 to 0.010) | 3.46E-12 | 0.612 (0.495 to 0.758) | 3.16E-03 | Yes |
| PLTP | 0.008 (0.004 to 0.011) | 1.35E-04 | 0.750 (0.659 to 0.853) | 4.83E-03 | Yes |
| CNTN4 | 0.008 (0.005 to 0.010) | 5.67E-09 | 0.650 (0.536 to 0.789) | 4.83E-03 | Yes |
| THBS2 | -0.009 (-0.012 to -0.005) | 2.38E-05 | 0.746 (0.653 to 0.852) | 4.96E-03 | No |
| NOTCH3 | 0.007 (0.004 to 0.010) | 1.70E-04 | 0.736 (0.640 to 0.847) | 4.98E-03 | Yes |
| ADAMTS13 | 0.004 (0.001 to 0.006) | 6.01E-03 | 0.659 (0.543 to 0.800) | 6.67E-03 | Yes |
| NCAM2 | 0.006 (0.004 to 0.009) | 4.85E-05 | 0.693 (0.583 to 0.822) | 6.67E-03 | Yes |
| TNFRSF4 | -0.006 (-0.010 to -0.003) | 2.36E-03 | 1.290 (1.141 to 1.458) | 9.35E-03 | Yes |
| ADAMTS8 | 0.012 (0.007 to 0.016) | 5.60E-06 | 0.810 (0.732 to 0.897) | 9.35E-03 | Yes |
| LBP | -0.012 (-0.017 to -0.006) | 6.15E-04 | 1.165 (1.077 to 1.262) | 2.61E-02 | Yes |
| APCS | -0.014 (-0.018 to -0.011) | 2.95E-14 | 1.351 (1.151 to 1.586) | 3.76E-02 | Yes |
| LEP | -0.039 (-0.045 to -0.032) | 1.49E-27 | 1.143 (1.064 to 1.228) | 4.16E-02 | Yes |

Directionally concordant indicates that the protein was either positively associated with DI-GM and inversely associated with GERD risk, or inversely associated with DI-GM and positively associated with GERD risk. All models were adjusted for the full covariate set used in the primary proteomic analyses.

**Supplementary Table 17 Baseline characteristics of the main analytical cohort and the proteomics analysis sample**

| **Characteristic** | **Main analytical cohort (n=133,915)** | **Proteomics analysis sample (n=15,506)** | **Absolute SMD** |
| --- | --- | --- | --- |
| Age, years | 55.71 ± 7.98 | 55.77 ± 8.09 | 0.008 |
| Female, n (%) | 70,770 (52.85%) | 8,206 (52.92%) | 0.001 |
| Body mass index, kg/m² | 26.76 ± 4.53 | 26.79 ± 4.51 | 0.006 |
| DI-GM score | 5.09 ± 1.93 | 4.54 ± 1.85 | 0.289 |
| Incident GERD, n (%) | 12,271 (9.16%) | 1,509 (9.73%) | 0.019 |

Data are presented as mean ± standard deviation or n (%). SMD, standardized mean difference; DI-GM, Dietary Index for Gut Microbiota; GERD, gastroesophageal reflux disease. The proteomics analysis sample was not generated by simple random sampling from the main analytical cohort.

**Supplementary Table 18 Random overlap expectation and robustness of pathway enrichment analyses**

Panel A. Random overlap expectation.

| **Metric** | **Value** |
| --- | --- |
| Background proteins analyzed | 2,910 |
| FDR-significant DI-GM-associated proteins | 1,057 |
| FDR-significant GERD-associated proteins | 19 |
| Expected overlap under random independence | 6.90 |
| Observed overlap | 13 |
| Observed-to-expected overlap ratio | 1.88 |
| Hypergeometric P value for observing at least 13 overlapping proteins | 0.0044 |

Panel B. Enrichment robustness across shared-protein query sets.

| **Query set** | **Query size** | **Significant GO:BP terms** | **Significant KEGG pathways** |
| --- | --- | --- | --- |
| All shared proteins, without directional pre-screening | 13 | 0 | 0 |
| Positive with DI-GM and inverse with GERD | 9 | 7 | 0 |
| Shared proteins inversely associated with DI-GM | 4 | 0 | 0 |

The 2,910 analyzed proteins were supplied as the enrichment background; the effective domain size was 2,760 after identifier mapping by g:Profiler. Statistical significance was assessed after multiple-testing correction across tested pathways. Enrichment of the nine proteins positively associated with DI-GM and inversely associated with GERD should be interpreted as exploratory because the all-shared-protein analysis was not significant.
